# Anisotropic conductivity modeling for tDCS in Parkinson’s disease using multidimensional diffusion MRI

**DOI:** 10.3389/fnhum.2026.1972676

**Published:** 2026-10-21

**Authors:** Santiago Osorio Jurado, Mikael Skorpil, Per Svenningsson, Rodrigo Moreno, Christoffer Olsson

**Affiliations:** 1 Department of Biomedical Engineering, The City College of New York, City University of New York, New York, NY, United States; 2 Department of Biomedical Engineering and Health Systems, KTH Royal Institute of Technology, Stockholm, Sweden; 3 Department of Molecular Medicine and Surgery, Karolinska Institutet, Stockholm, Sweden; 4 Department of Clinical Neuroscience, Karolinska Institutet, Stockholm, Sweden; 5 Department of Neurobiology, Care Sciences and Society, Karolinska Institutet, Stockholm, Sweden

**Keywords:** diffusion MRI, electric field modeling, finite element method, Parkinson disease, q-space trajectory imaging, transcranial direct current stimulation

## Abstract

**Introduction:**

Predictions of the electric field (E-field) induced by transcranial direct current stimulation (tDCS) depend on how brain conductivity is modeled. White matter anisotropy is conventionally estimated from single-shell diffusion tensor imaging (DTI). Multidimensional diffusion MRI (MD-dMRI), specifically q-space trajectory imaging (QTI), instead gives a mean tensor expected to carry less kurtosis bias. Our primary question was whether replacing the conventional single-shell tensor with this mean tensor would change the predicted E-field.

**Methods:**

We built, to our knowledge, the first MD-dMRI tDCS conductivity model and compared it against DTI and isotropic models in 29 participants (12 with Parkinson’s disease and 17 controls) across four montages, with the same mesh, electrodes, and solver.

**Results:**

The two anisotropic models differed mainly in tensor orientation (18–25 degrees across the white matter), with E-field magnitudes differing by 6.6% or less. E-fields were not significantly different between patients and controls in any region or montage (which was an exploratory, underpowered comparison). Whole-brain E-field correlated with MR elastography stiffness (partial *r* = 0.58) but attenuated to non-significance once cerebrospinal fluid (CSF) morphology was accounted for (*r* = 0.06 to 0.09).

**Discussion:**

With no ground-truth E-field or conductivity available, the study establishes the feasibility of the MD-dMRI model and characterizes E-field sensitivity rather than improved dosimetry accuracy. The choice of diffusion tensor is second order for the predicted E-field, which is primarily influenced by individual anatomy. For Parkinson’s disease, modeling efforts should focus on CSF- and atrophy-aware head models and dose normalization, rather than a more complex diffusion tensor.

## Highlights

- Individual anatomy, more than the conductivity tensor, governs the predicted E-field.
- A first tDCS head model from MD-dMRI (QTI).
- MD-dMRI and single-shell DTI E-fields differed by 6.6% or less.
- The anisotropic models differed mainly in tensor orientation.

## 1 Introduction

tDCS is currently under investigation as a non-invasive therapy in Parkinson’s disease (PD), where it aims to modulate cortical and network excitability (Fregni et al., 2006; Broeder et al., 2015; Lefaucheur et al., 2017). Its effects depend on the E-field delivered to the target (Nitsche and Paulus, 2000), with a non-linear dose–response (Jamil et al., 2017); however, the E-field cannot be measured directly, and it varies substantially across individuals since it depends on head anatomy and on the electrical conductivity of brain tissue (Laakso et al., 2015; Huang et al., 2017). Because the dose–response relationship is non-linear, an inaccurate E-field estimate can place a subject at the wrong point on that curve, which is one proposed contributor to the inconsistent clinical effects reported in tDCS trials for PD (Elsner et al., 2016). Personalized finite-element head models estimate the E-field from individual anatomy (Thielscher et al., 2015; Windhoff et al., 2013; Saturnino et al., 2019a).

White matter (WM) is electrically anisotropic, and its conductivity tensor is conventionally derived from single-shell DTI through a volume-normalized mapping, which translates the shape and orientation of the water diffusion tensor into the conductivity tensor (Tuch et al., 2001; Rullmann et al., 2009; Güllmar et al., 2010). However, the single-shell tensor is a biased estimate of the macroscopic tensor. A mono-exponential fit disregards non-Gaussian diffusion (Jensen et al., 2005), making the estimate dependent on the *b*-value and susceptible to errors caused by free water (Pasternak et al., 2009). Single-shell WM metrics vary widely across studies in PD (Atkinson-Clement et al., 2017), so the anisotropy derived from a DTI model is itself uncertain. Modeling a kurtosis term alongside diffusion yields a *b*-value-independent tensor estimate (Veraart et al., 2011).

MD-dMRI, in particular QTI with tensor-valued encoding, estimates the macroscopic mean tensor alongside the covariance of the intra-voxel tensor distribution, placing the non-Gaussian variance in the covariance term rather than in the mean (Westin et al., 2016; Topgaard, 2017; Szczepankiewicz et al., 2019). This extends the benefit of modeling the kurtosis separately, as in diffusional kurtosis imaging, to tensor-valued encoding. The resulting mean tensor is therefore expected to be less kurtosis-biased, and the decomposition has validated biophysical meaning and clinical relevance in tumor and WM disease (Szczepankiewicz et al., 2016; Nilsson et al., 2020; Andersen et al., 2020). Unlike conductivity tensor imaging, where conductivity is derived from an extracellular water diffusion tensor scaled by high-frequency electrical properties and volume fractions (Katoch et al., 2023), MD-dMRI has not, to our knowledge, been used to build a tDCS conductivity model, and it is unknown whether a less kurtosis-biased tensor changes the predicted E-field enough to matter for dosimetry.

Here, we build a tDCS conductivity model from the MD-dMRI mean tensor and ask whether replacing the conventional single-shell tensor with it changes the predicted E-field. Using the cohort of Olsson et al. (2025), 12 patients with PD and 17 controls scanned with both single-shell and MD-dMRI acquisitions, we compare three conductivity models, namely, isotropic, single-shell DTI, and MD-dMRI, under an identical head model, electrode set, and solver. Our primary question is as follows: for a given subject and montage, how much does the E-field change when only the conductivity model changes? As a secondary, exploratory question, we ask whether patients and controls differ in the modeled E-field. Additionally, we examine the E-field’s association with the brain’s mechanical properties, measured by magnetic resonance elastography (MRE) in the same cohort. In this study, we analyze the feasibility of building an MD-dMRI head model and the sensitivity of the predicted E-field to the choice of diffusion tensor.

## 2 Methods

### 2.1 Study design

We compared tDCS E-field predictions across three tissue conductivity models in one cohort of patients with PD and healthy controls (HC). The three models shared an identical head mesh, electrode configuration, and finite element solver and differed only in how conductivity was assigned to brain tissue. The first model used a single isotropic value per tissue. The second derived an anisotropic conductivity tensor from a conventional single-shell diffusion tensor acquisition. The third derived the tensor from the macroscopic mean diffusion tensor estimated with MD-dMRI (QTI).

The study addressed two questions. The primary question was as follows: for a given subject and montage, how much does the predicted E-field change when the conductivity model changes? Because every subject contributes to all three models, we treated this as a paired comparison. The secondary question was between groups: do patients and controls differ in the E-field metrics produced by using the MD-dMRI mean diffusion tensor? Since the cohort was not powered for this comparison, the results are exploratory. Figure 1 gives an overview of the modeling pipeline and the four electrode montages.

**Figure 1:**
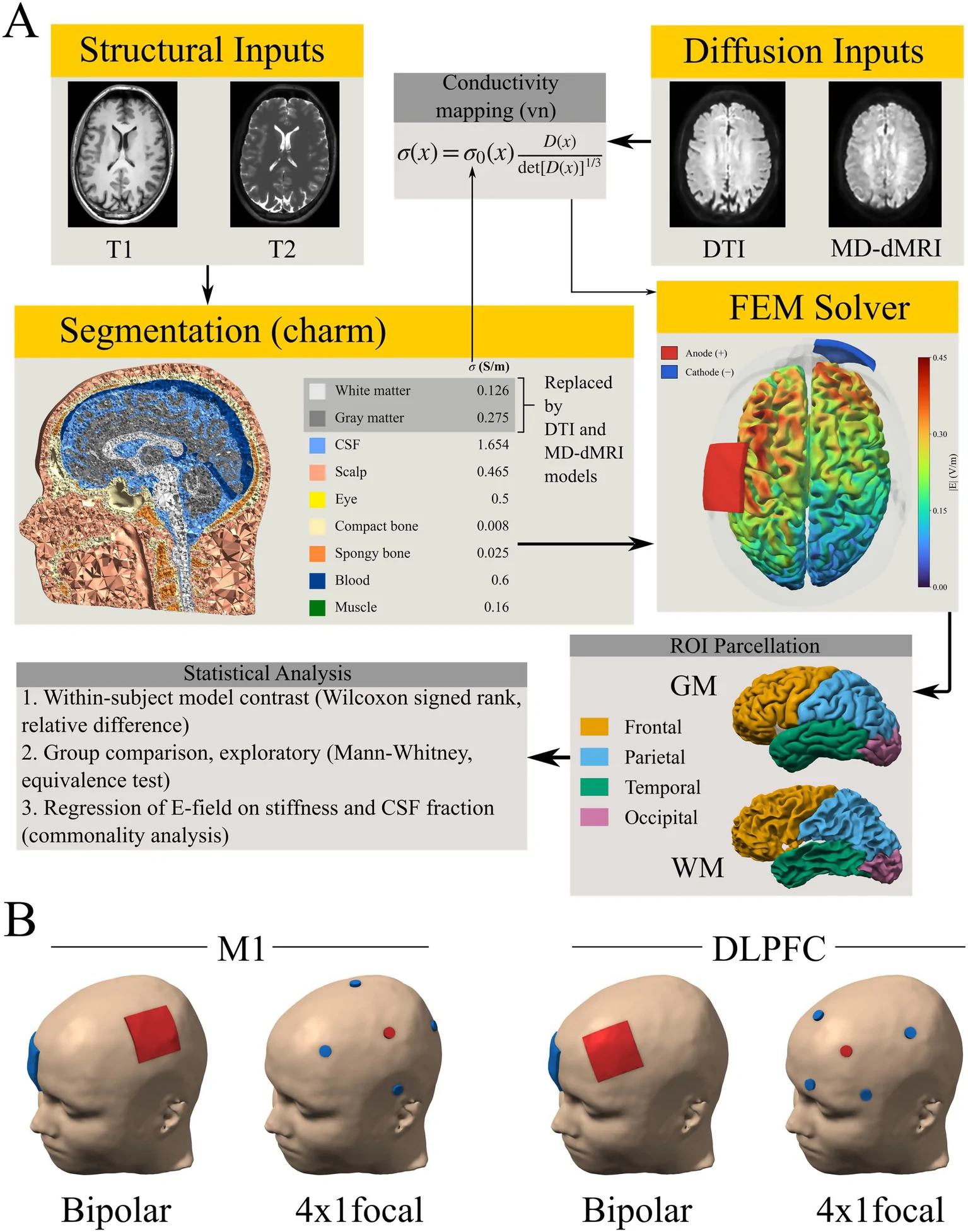
Overview of the modeling pipeline and electrode montages. **(A)** For each participant, the structural and diffusion MR images feed a single head segmentation and finite element mesh. The three conductivity models (isotropic, DTI, and MD-dMRI) share that mesh and the same E-field solver and differ only in the conductivity assigned to brain tissue. **(B)** The four tDCS montages: bipolar pads and 4 × 1 focal arrays at the M1 (primary motor cortex) and DLPFC (dorsolateral prefrontal cortex) targets. In each head, the red electrode is the anode, and the blue electrodes are the cathodes. Head images are the SimNIBS example head, Ernie, not a study participant.

### 2.2 Participants and data

We used the dataset described in Olsson et al. (2025). The cohort comprised 29 participants: 12 with PD, assessed in the ON medication state (three women and nine men; age 63 ± 9 years, range 47–78), and 17 HC (three women and 14 men; age 60 ± 6 years, range 50–78). The full demographic and clinical characterization is reported by Olsson et al. (2025). All participants gave written informed consent, and the original study was approved by the Swedish Ethical Review Authority (Dnr. 2022–03209-02). Our analysis was a secondary use of these data, covered by that approval. We analyzed the full cohort and excluded no participants.

All scans were acquired on a Philips Ingenia CX 3 T system (Philips Healthcare, Best, the Netherlands) with a 32-channel head coil. The session provided a T1-weighted image (Turbo Field Echo, TR/TE 6.7/3 ms, 1 mm isotropic, full-head FOV), a T2-weighted image (TR/TE 3000/280 ms, 1 mm isotropic), a single-shell diffusion acquisition for the DTI model, and a multidimensional diffusion (QTI) acquisition for the MD-dMRI model. The QTI acquisition followed Topgaard (2017) and Szczepankiewicz et al. (2019), using linear and spherical *b*-tensor encoding at five *b*-values (0, 100, 700, 1,400, and 2,000 s/mm^2^), TR/TE 4000/111 ms, 2.5 mm isotropic, in 6.5 min. The single-shell diffusion acquisition comprised 80 directions at *b* = 1,500 s/mm^2^ with one *b* = 0 volume, TR/TE = 2658/83 ms, isotropic = 2.0 mm resolution, and multiband factor = 3. This single-shell sequence was acquired in the same session but is not among the acquisitions reported by Olsson et al. (2025). The two diffusion acquisitions came from separate scans in the same session, so the DTI and MD-dMRI conductivity models come from separate acquisitions rather than two reconstructions of the same raw data.

### 2.3 Head model

We generated a tetrahedral head model for each subject with the charm pipeline in SimNIBS 4.6 (Thielscher et al., 2015; Puonti et al., 2020). Charm segments the T1- and T2-weighted images into tissue classes and produces a conforming finite element mesh. The E-field simulation assigns an isotropic conductivity to nine of these classes: WM, gray matter (GM), CSF, scalp, eyes, compact bone, spongy bone, blood, and muscle. We ran charm with its default settings and inspected every segmentation visually. No manual corrections were applied.

### 2.4 Conductivity models

Both diffusion acquisitions were corrected as part of the cohort’s preprocessing, before any tensor was fitted. The single-shell acquisition was corrected for susceptibility distortion, eddy currents, and subject motion with FSL topup and eddy (Andersson et al., 2003; Andersson and Sotiropoulos, 2016), and the gradient directions were rotated accordingly. The QTI acquisition underwent the same three corrections, applied to the linear and spherical series separately, with a synthetic reverse-phase-encoded *b* = 0 image supplying the susceptibility estimate (Synb0-DisCo; Schilling et al., 2019, 2020). Before the covariance fit, we co-registered the two series with an affine transform (elastix; Klein et al., 2010) and masked the result by intensity threshold. All parameters are listed in Supplementary Table S1.

The three models differ only in the conductivity assigned to WM and GM. Every other compartment received the same isotropic conductivity in all three models, and the isotropic model used isotropic conductivities throughout. We used the SimNIBS 4.6 default scalar conductivities (shown in Figure 1), with WM and GM set to 0.126 and 0.275 S/m, respectively (Wagner et al., 2004). The isotropic model serves as a baseline with no anisotropy. The primary comparison is between the two anisotropic models, with the conventional single-shell DTI tensor acting as the reference for the MD-dMRI tensor.

Both anisotropic models map a diffusion tensor to a conductivity tensor with the volume-normalized rule (Güllmar et al., 2010; Rullmann et al., 2009), implemented in SimNIBS as anisotropy_type = ‘vn’:

$$\sigma (x)=\sigma_{0}(x)\;D(x)/\det{\left[D(x)\right]}^{1/3}.$$

where D(x) is the voxel diffusion tensor, and $\sigma_{0}(x)$ is the isotropic tissue conductivity. The conductivity tensor inherits the eigenvectors of D , and the determinant term fixes the geometric mean of its eigenvalues to one, so only the shape and orientation of D survive the mapping, and the scale of each tissue is set by $\sigma_{0}$ . We left both SimNIBS bounding parameters at their defaults from version 4.6, identical for both models.

For the DTI model, we fitted the single-shell diffusion tensor with FSL dtifit by ordinary least squares (Jenkinson et al., 2012) and passed it to dwi2cond (Opitz et al., 2011) for its T1 coregistration and reorientation, using a 12-degree-of-freedom (DOF) affine, so the DTI model shares the registration class of the MD-dMRI model. Leaving dwi2cond otherwise at its defaults keeps the DTI model aligned with standard practice.

For the MD-dMRI model, the diffusion tensor was the QTI macroscopic mean tensor (Westin et al., 2016; Topgaard, 2017). We fitted the QTI signal with the diffusion tensor distribution covariance model, using the MD-dMRI framework (Nilsson et al., 2018), matching the processing applied to this cohort by Olsson et al. (2025). The QTI signal was spatially smoothed before fitting (isotropic Gaussian, σ=0.7 voxels) to reduce noise and stabilize the covariance fit. The covariance model estimates more parameters per voxel than a single-shell tensor, and without smoothing it was non-positive-definite in a median of 11% of brain voxels, with a noise-inflated WM anisotropy (near 15:1 in the most anisotropic voxels, above the 10:1 physiological ceiling, against near 4:1 when smoothed). We kept the smallest smoothing kernel at which fewer than 5% of voxels in any participant were non-positive-definite. We repeated the fit, conductivity mapping, and M1 bipolar simulation at kernels of 0, 0.4, 0.5, and 1.4 voxels (Supplementary Table S2). The smoothing therefore trades spatial resolution for a physically plausible tensor. Its consequence for the between-model (MD-dMRI versus (vs.) DTI) E-field magnitude comparison is taken up in Section 4.3. The DTI tensor received no comparable smoothing, so the two anisotropic inputs differ in resolution and smoothing as well as in estimation method. Therefore, the difference in E-field magnitude between the two models, measured as its 95th percentile (*p*_95_ |E|; Section 2.8), cannot be attributed to the estimation method alone, as it is confounded by these acquisition and smoothing differences. Our claims about how the two tensor estimates differ rest instead on their orientation. The mean tensor is the first cumulant of that fit, the macroscopic mean diffusion tensor, estimated jointly with the covariance of the intra-voxel tensor distribution. Because the non-Gaussian variance is carried by the covariance term rather than absorbed into the mean, the mean tensor targets the same macroscopic tensor as the single-shell fit with reduced kurtosis bias (Westin et al., 2016; Veraart et al., 2011; Jensen et al., 2005). To obtain the MD-dMRI-oriented macroscopic mean tensor required for conductivity mapping, we re-fitted the covariance model to the corrected linear and spherical encoding series, changing only the fit output used, not the upstream preprocessing. The encoding was carried through that motion and distortion correction, so the reconstructed orientation is in register with the corrected data. We then supplied that tensor to the same volume-normalized (‘vn’) mapping and solver used in the DTI model. The two anisotropic models are therefore identical in mapping and solver, differing only in the diffusion tensor and its acquisition. A voxel was set to isotropic before conductivity mapping when the smallest eigenvalue of the mean diffusion tensor was at or below 0.001 μm^2^/ms, a non-positive-definite fit, or when its mean diffusivity was below 0.2 μm^2^/ms, far below the diffusivity of brain tissue. This rule affected a median of 1.6% of the voxels in the diffusion brain mask (interquartile range 0.6 to 2.6%, maximum 4.8%), concentrated at CSF borders and low-signal edges. These voxels received the isotropic tissue conductivity and contributed neither anisotropy nor orientation.

### 2.5 Registration and tensor reorientation

The conductivity tensors are defined in diffusion space and must be moved into the head model’s anatomical space before they can be assigned to mesh elements. In each subject, a non-linear warp added spurious deformation without improving alignment, so we chose an affine transformation. We registered the MD-dMRI model’s S0 image to the charm T2 with a 12-DOF affine (FSL FLIRT, mutual information; Jenkinson and Smith, 2001; Jenkinson et al., 2002), initialized from a rigid pre-alignment and rejected if degenerate, and applied that transform to every map.

Moving a tensor requires reorienting it, not merely resampling its components (Alexander et al., 2001). We warped the three eigenvalues independently as scalar maps (trilinear) and carried the orientation frame with vecreg, anchoring the principal axis to the registered principal eigenvector. After interpolation, the eigenvalue maps were sorted and paired once again with the frame by magnitude, so a boundary voxel where two maps cross cannot misassign an eigenvalue to an eigenvector. We validated each subject’s registration against the known fiber orientation of two reference structures segmented from each subject’s own anatomy: left–right across the corpus callosum and superior–inferior through the midbrain, which carries the cerebral peduncles. Each registration also passed automated field-of-view containment and S0-to-T1 edge-alignment checks and agreed with the fractional anisotropy (FA) registration to T1 provided with the cohort.

The angle between the DTI and MD-dMRI tensors confounds the estimation model with the difference between the two scans. Within the QTI acquisition, we therefore fitted a diffusion tensor with dtifit, to its 43 linear-encoded volumes and, as a sensitivity analysis, to its *b* = 2,000 s/mm^2^ shell with the *b* = 0 volumes and compared each with the mean diffusion tensor of the covariance fit. Scan, resolution, preprocessing, smoothing, and mask were identical, and both tensors were compared in the QTI space. The covariance model cannot be fitted to linear encoding alone, so its mean tensor is estimated from the spherical series as well. This control is reported with the results.

### 2.6 Electrode montages

We simulated four montages, chosen to cover the two cortical targets most used in PD tDCS and to contrast conventional bipolar pads with 4 × 1 focal arrays (Table 1). Two were bipolar: a primary motor cortex (M1) bipolar montage (anode over C3, cathode over Fp2) and a dorsolateral prefrontal cortex (DLPFC) bipolar montage (anode over F3, cathode over Fp2), each with 5 × 5 cm pads at 2 mA. Two were 4 × 1 focal montages (Datta et al., 2009): an M1 montage with the anode at C3 and cathodes at Cz, F3, T7, and P3, and a DLPFC montage with the anode at F3 and cathodes at Fp1, Fz, C3, and F7. In each 4 × 1 focal montage, the anode delivered 2 mA, and the four cathodes each carried −0.5 mA.

**Table 1:** Electrode montages simulated.

Target	Electrode type	Anode	Cathode(s)	Current
M1	Bipolar (5 × 5 cm)	C3	Fp2	+2 mA anode; −2 mA cathode
M1	4 × 1 focal	C3	Cz, F3, T7, P3	+2 mA anode; −0.5 mA each cathode
DLPFC	Bipolar (5 × 5 cm)	F3	Fp2	+2 mA anode; −2 mA cathode
DLPFC	4 × 1 focal	F3	Fp1, Fz, C3, F7	+2 mA anode; −0.5 mA each cathode

The four montages span two cortical targets (primary motor cortex, M1; dorsolateral prefrontal cortex, DLPFC) crossed with two electrode types: a bipolar configuration (5 × 5 cm rectangular pads, 25 cm^2^, 0.080 mA/cm^2^ at 2 mA) and a 4 × 1 focal array of 12 mm diameter disc electrodes. For each subject, all montages used the same head mesh, finite-element solver, and 2 mA total current, and the two electrode types at each target shared the anode position. Electrode labels are 10–10 EEG positions.

### 2.7 Regions of interest

The analysis covered the four GM and four WM lobes in each hemisphere, 16 regions in all, with the whole brain as a summary region. We kept the hemispheres separate because the montages are left-lateralized. GM and WM were parcellated with FreeSurfer 7.2 (Fischl, 2012) as part of the cohort’s preprocessing (Olsson et al., 2025), grouping the Desikan–Killiany regions (Desikan et al., 2006) into frontal, parietal, temporal, and occipital lobes and taking the WM lobes from the wmparc segmentation. We registered these lobes to the charm T1 with a rigid transform, resampled them by nearest neighbor, and gave each mesh element the label at its barycenter. Tissue class came from charm: A GM region was the set of charm GM elements inside the FreeSurfer GM lobe, and a WM region was the set of charm WM elements inside the wmparc lobe, so each region carries only elements of its own tissue.

All regions of interest (ROIs) were defined anatomically, from each subject’s own FreeSurfer segmentation, before the E-field was computed. No region was selected or adjusted based on the E-field it contained.

### 2.8 E-field metrics and statistics

For each subject, montage, and conductivity model, we solved the E-field on the head mesh (Windhoff et al., 2013; Saturnino et al., 2019a) and summarized its magnitude within each ROI over the GM and WM elements. Specifically, we report two E-field metrics per region. The 95th percentile of |E| is the E-field metric for the model comparison. It summarizes the regional E-field without being inflated by the outlier elements (Huang et al., 2017). The median of |E| is used for the age-adjusted group comparison and the stiffness correlation. We also interpolated the E-field of each model onto the cortical middle surface and report three surface metrics per region and montage (Supplementary Table S4). For the normal component of the E-field (nE), we report the change in the region mean and the mean absolute difference across surface points, both relative to the mean |nE| of the DTI model (Kimura et al., 2026). For |E|, we report the root mean square of the difference across surface points, relative to the root mean square |E| of the DTI model (Mosayebi-Samani et al., 2025). Following Kimura et al. (2026), we categorize relative differences between models as moderate if they fall between 10 and 25%, and as large if they exceed 25%. Differences below 10% are considered small.

For the primary within-subject comparison, we tested in each region and montage whether the E-field metric differed between conductivity models with the Wilcoxon signed-rank test, using the matched-pairs rank-biserial correlation as the effect size (Kerby, 2014). For each region, we also summarized the within-subject difference in $p_{95}$ |E| (MD-dMRI minus DTI) at the cohort median, with a 95% confidence interval from a percentile bootstrap (10,000 subject-level resamples, Table 2). For the exploratory comparison between groups, we compared patients and controls under the MD-dMRI model. Following Olsson et al. (2025), we adjusted each E-field metric for a linear age effect by ordinary least squares and compared the residuals between groups with the Mann–Whitney U test, reporting Cohen’s *d*. We made no correction for sex, given the small number of women in each group. Our primary analysis consists of the 16 paired tests in the lobar regions under the M1 bipolar montage ( $p_{95}$ |E|). The other three montages and the median metric are secondary. The comparison between groups is exploratory. Additionally, we asked whether orientation divergence between the two anisotropic tensors drives the difference in the E-field between the models. Across all subjects and WM regions, where the principal direction is well defined, we correlated, for each region, the angle between the eigenvector of the largest eigenvalue of the two tensors (the principal eigenvector, V1) with the absolute percentage difference between the two models, with reference to the DTI model (Δ = 100 |*E*_MD-dMRI_ − *E*_DTI_| / *E*_DTI_) in $p_{95}$ |E|. The V1 angle is the acute angle between the DTI and QTI mean tensor principal eigenvectors (*θ* = arccos(|*v*_DTI_ · *v*_QTI_|), the absolute value resolving the sign ambiguity), computed per voxel and reported as the cohort median of each subject’s ROI median. Before correlating, we centered each region on its mean across each subject, so the correlation captures the variation within ROI rather than differences between regions. We obtained the 95% confidence interval from a subject-clustered bootstrap that resampled the 29 subjects with replacement, since each subject contributes to several correlated regions.

**Table 2:** Region-wise paired comparison of the predicted E-field, MD-dMRI vs. DTI, for the 16 regions under the M1 bipolar montage.

Region	Δ*p*_95_ |*E*| (*V*/*m*)	95% CI	Rel. diff. (%), mean [SD]	Rel. diff. (%), min to max	*r_rb_*	*q*
Frontal GM L	0.0011	[0.0007, 0.0030]	0.6 [1.0]	−0.9 to 4.2	0.694	0.001
Frontal GM R	0.0028	[0.0018, 0.0041]	0.9 [1.3]	−0.6 to 6.7	0.862	<0.001
Parietal GM L	0.0042	[0.0027, 0.0050]	1.3 [0.7]	0.0 to 3.0	1.000	<0.001
Parietal GM R	0.0005	[0.0002, 0.0018]	0.5 [1.2]	−2.1 to 4.3	0.476	0.028
Temporal GM L	0.0012	[0.0001, 0.0017]	0.4 [0.7]	−0.9 to 2.5	0.584	0.007
Temporal GM R	−0.0005	[−0.0013, 0.0003]	−0.1 [1.0]	−2.1 to 2.6	−0.191	0.381
Occipital GM L	0.0013	[0.0003, 0.0021]	0.8 [1.0]	−0.9 to 3.1	0.779	<0.001
Occipital GM R	0.0001	[−0.0002, 0.0009]	0.4 [1.4]	−2.1 to 5.0	0.283	0.203
Frontal WM L	−0.0258	[−0.0340, −0.0226]	−5.2 [1.6]	−9.1 to −1.8	−1.000	<0.001
Frontal WM R	−0.0246	[−0.0297, −0.0191]	−4.6 [1.4]	−8.3 to −2.2	−1.000	<0.001
Parietal WM L	−0.0221	[−0.0281, −0.0194]	−5.2 [1.9]	−8.3 to −1.0	−1.000	<0.001
Parietal WM R	−0.0121	[−0.0146, −0.0106]	−4.3 [1.4]	−7.0 to −2.0	−1.000	<0.001
Temporal WM L	−0.0262	[−0.0316, −0.0238]	−6.4 [1.7]	−9.1 to −3.3	−1.000	<0.001
Temporal WM R	−0.0098	[−0.0109, −0.0074]	−3.6 [1.4]	−6.2 to −0.2	−1.000	<0.001
Occipital WM L	−0.0155	[−0.0172, −0.0121]	−5.2 [1.4]	−7.7 to −2.6	−1.000	<0.001
Occipital WM R	−0.0076	[−0.0096, −0.0069]	−4.4 [1.4]	−6.8 to −1.7	−1.000	<0.001

GM, gray matter lobe; WM, white matter lobe; L and R, left and right hemisphere. Δp_95_ |*E*| (*V*/*m*) is the within-subject difference in the 95th-percentile E-field magnitude (MD-dMRI minus DTI) at the cohort median, with a percentile-bootstrap 95% confidence interval. The relative difference is that difference as a percentage of the DTI value, computed per participant and reported as the mean with its standard deviation and the range across participants. *r_rb_* is the matched-pairs rank-biserial effect size (Wilcoxon signed-rank, paired); positive *r_rb_* indicates MD-dMRI exceeds DTI, negative *r_rb_* indicates the opposite. *q* is the Benjamini–Hochberg FDR-adjusted *p*-value across the 16 regions; *q* < 0.001 is shown as <0.001, and *q* < 0.05 indicates significance.

The false discovery rate across regions was controlled through the Benjamini–Hochberg procedure within each montage and E-field metric for the model comparison (Benjamini and Hochberg, 1995), treating a Benjamini–Hochberg-adjusted *q* < 0.05 as significant. The PD vs. HC comparison is exploratory, so we report uncorrected *p*-values for it. With *n* = 29 paired observations, a two-sided paired test at alpha = 0.05 has 80% power to detect a within-subject effect of $d_{z}$ about 0.54 (a non-central-*t* approximation to the Wilcoxon signed-rank test). The between-group comparison, with 12 patients and 17 controls, has 80% power only for a group difference of Cohen’s *d* = 1.1, and approximately 26, 64, and 176 participants per group would be needed to detect effects of *d* = 0.8, 0.5, and 0.3 at the same power. It cannot exclude moderate effects, which is the second reason we treat it as exploratory. We also report it as an equivalence test, with two one-sided tests against equivalence bounds of 10 and 25% of the HC mean.

Diffusion preprocessing and registration used FSL 6.0.7.22. The QTI mean tensor was fitted with the MD-dMRI framework in MATLAB R2026a (The MathWorks, Natick, MA, USA). Statistical analyses were run in Python 3.11.14 with NumPy 2.3.0 (Harris et al., 2020) and SciPy 1.17.1 (Virtanen et al., 2020).

### 2.9 Association with mechanical properties

As a secondary analysis, we related the predicted E-field to the cohort’s mechanical measures, testing whether whole-brain median |E| covaried with MRE stiffness (|G*|, the magnitude of the complex shear modulus, computed whole-brain excluding ventricles, and CSF) across all 29 subjects, following the correlation approach of Olsson et al. (2025). We computed the partial Pearson correlation between median |E| and stiffness, controlling for group (PD/HC) as a covariate, and adjusted the correlation *p*-values with the Benjamini–Hochberg false discovery rate procedure, reported as *q* throughout (Benjamini and Hochberg, 1995), as in the ROI analyses. Then, we added age as a second covariate, since both stiffness and head morphology change with age and separately added CSF volume and the CSF fraction, computed as CSF volume divided by the sum of CSF, WM, and GM, both from the charm tissue segmentation. The CSF covariates test whether the E-field and stiffness relationship is driven by CSF morphology rather than a direct effect of tissue stiffness, since current shunts through the highly conductive CSF and less E-field reaches the cortex in subjects with larger CSF volumes (Indahlastari et al., 2020). Additionally, we computed the partial correlation of |E| with CSF fraction controlling for stiffness, and the collinearity between stiffness and CSF fraction, which we report descriptively, without false-discovery correction. The correlation was repeated under all three conductivity models, and we report the MD-dMRI model as representative. Because that collinearity makes the stepwise partial correlations hard to interpret, we also fitted a joint ordinary least squares regression of whole-brain median |E| on stiffness, CSF fraction, age, and group, with standardized coefficients, 95% confidence intervals, and variance inflation factors, and partitioned the variance the two collinear predictors explain by commonality analysis (Table 3). Finally, as an input check, we correlated mean diffusivity with stiffness to confirm that our diffusion inputs reproduce the negative association reported by Olsson et al. (2025), whose stiffness maps we used directly. We did not report the stiffness-microstructure correlations as findings of this study, since they re-derive Olsson et al. on the same data.

**Table 3:** Whole-brain median |E| (MD-dMRI model, M1 bipolar montage) against MRE stiffness, CSF fraction, age, and group.

Panel A. Regression coefficients				
Predictor	*β*	95% CI	*p*	VIF
MRE stiffness	0.23	[−0.44, 0.89]	0.490	4.9
CSF fraction	−0.82	[−1.48, −0.17]	0.016	4.8
Age	0.42	[−0.10, 0.94]	0.109	3.0
Group (PD vs. HC)	0.09	[−0.27, 0.45]	0.609	1.4
Model *R*^2^	0.49			

Panel B. Commonality analysis of stiffness and CSF fraction, adjusted for age and group			
Effect	Coefficient	95% CI	Percent
Unique to stiffness	0.012	[<0.001, 0.147]	3.1
Unique to CSF fraction	0.168	[0.010, 0.420]	42.3
Common to stiffness and CSF fraction	0.218	[0.044, 0.443]	54.6
Total	0.398	[0.168, 0.702]	100.0

Panel A is the multiple regression on all four predictors. Predictors are standardized, so each coefficient is the change in |E| in standard deviations per standard deviation of the predictor, written *β*. VIF is the variance inflation factor. Panel B partitions the variance that stiffness and CSF fraction can explain, after adjusting for age, and group, into the part unique to each and the part common to both, with each as a percent of that partition’s total. Intervals are from the *t* distribution in Panel A and a percentile bootstrap in Panel B.

## 3 Results

### 3.1 Participants

All 29 participants completed the structural, DTI, and MD-dMRI acquisitions and produced a head model that passed visual inspection of its segmentation. In every subject, the reoriented principal eigenvector agreed with the corpus callosum (left–right) and cerebral peduncle (superior–inferior) structural references.

### 3.2 Conductivity model comparison (MD-dMRI vs. DTI)

The two anisotropic models produced very similar E-fields. Under the M1 bipolar montage, the within-subject difference in the 95th percentile E-field magnitude ( $p_{95}$ ∣E∣ ) was small (below 10%), yet statistically resolvable in 14 of the 16 regions (Wilcoxon signed-rank, Benjamini–Hochberg-corrected within the montage), and the difference changed sign with tissue type. In GM, MD-dMRI slightly exceeded DTI, whereas in the WM lobes, MD-dMRI was below DTI by a larger margin and that reduction was larger in the left hemisphere, under the anode. On the cortical middle surface, the normal component of the E-field diverged more than its magnitude, although both remained below 10% (Supplementary Table S4). The magnitudes, relative differences, and effect sizes per region are given in Table 2, and the between-model orientation difference is reported in Section 3.5.

The same sign pattern, MD-dMRI above DTI in GM and below in WM, was consistent across all four montages, without exception in the WM lobes and in 28 of the 32 combinations of GM lobe and montage (Figure 2; Supplementary Table S3). Significance was reached in 15 of 16 under DLPFC bipolar, 16 of 16 under M1 4 × 1 focal, and 14 of 16 under DLPFC 4 × 1 focal.

**Figure 2:**
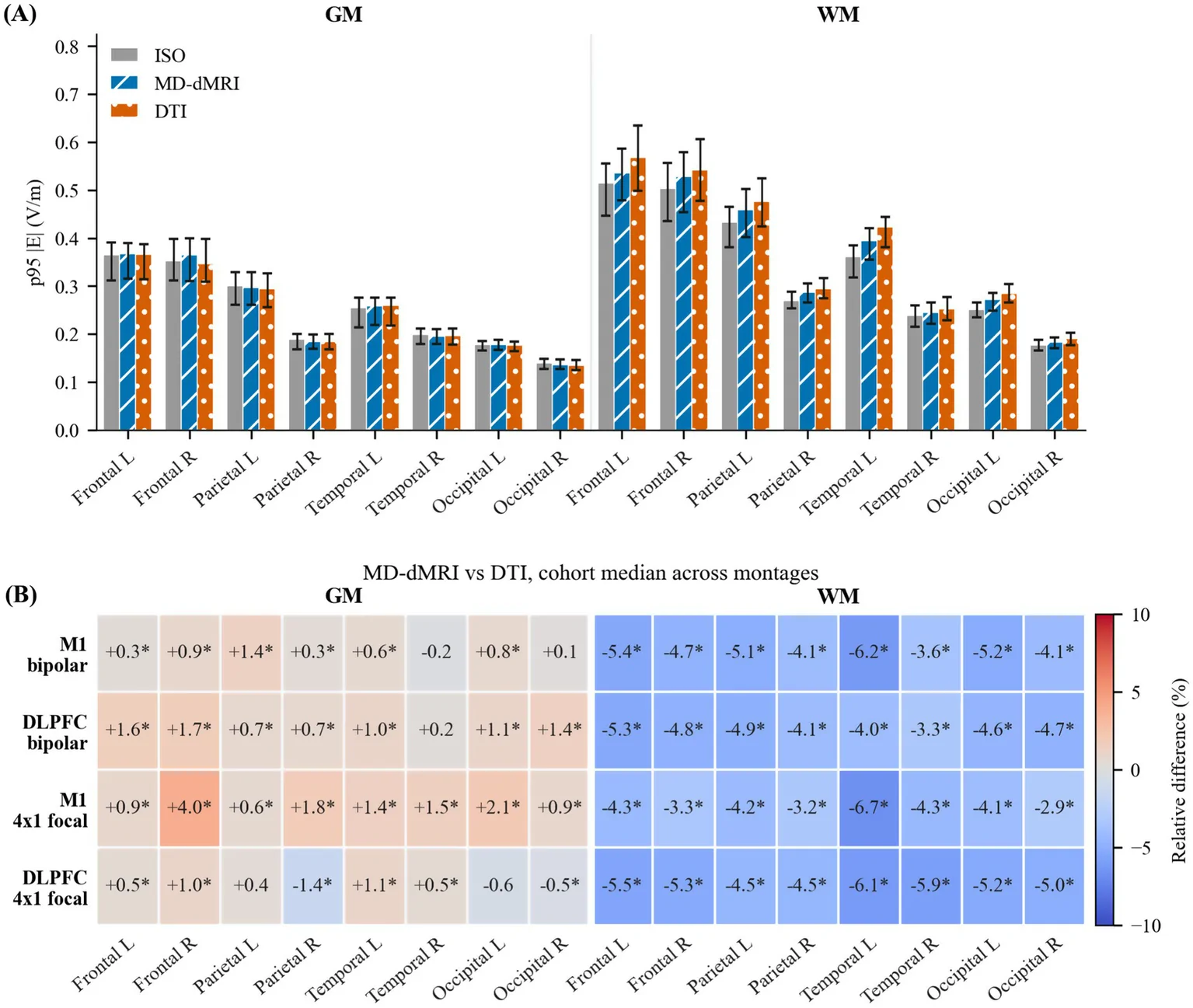
Predicted E-field per region for the three conductivity models and the MD-dMRI vs. DTI difference across montages. **(A)** Cohort median (bars) and interquartile range (whiskers) of p95 |E| in each region for the isotropic (ISO), MD-dMRI, and DTI models under the M1 bipolar montage. **(B)** Cohort median of the relative difference in p95 |E| (MD-dMRI minus DTI, as a percentage of DTI) in each of the 16 regions under each of the four montages, per montage (rows) and region (columns). Asterisks mark *q* < 0.05 from a two-sided Wilcoxon signed-rank test with Benjamini–Hochberg control of the false discovery rate across the 16 regions within each montage. The color scale is centered at zero and clipped at plus or minus 10% (red, MD-dMRI above DTI; blue, MD-dMRI below DTI). Exact values and effect sizes are given in Table 2.

### 3.3 Model difference relative to E-field magnitude

The absolute differences between models were small (Figure 2A). In GM lobes, the three models lay within 4.9% of one another. In the WM lobes, the two anisotropic models sat above the isotropic reference, MD-dMRI by 2.8 to 12.9% and DTI by 6.8 to 18.2%, with MD-dMRI below DTI, and the between-model differences stayed small relative to both the E-field magnitude and the between-subject interquartile spread. On the GM surface of a representative subject, the median absolute model difference was 0.0026 V/m, 1.8% of the median ∣E∣ (Figure 3). Most of the GM and WM volume lay within 10% of the corresponding DTI value (Figure 3C).

**Figure 3:**
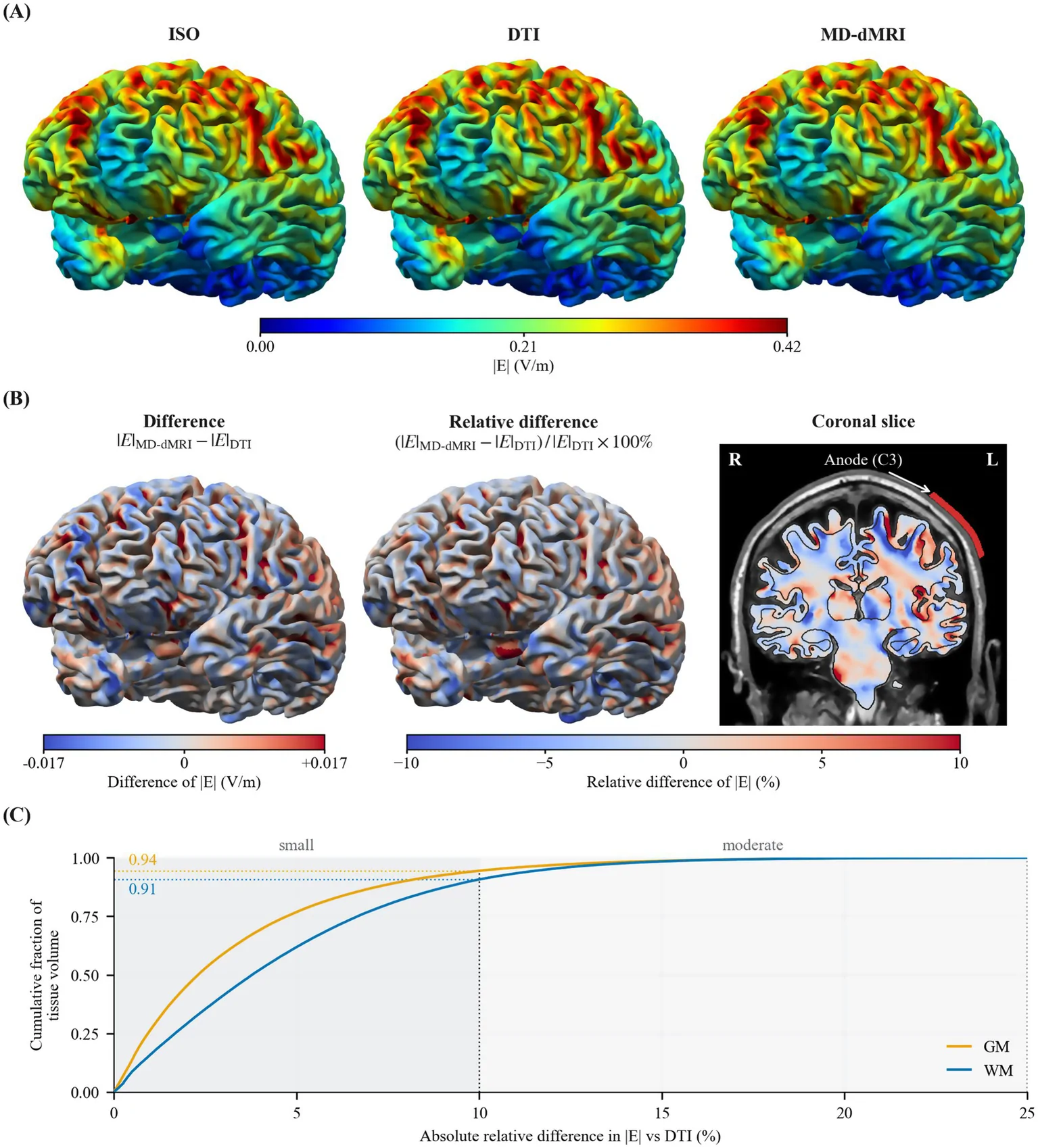
Predicted E-field on the cortical surface for each model and the difference between the two anisotropic models for the M1 bipolar montage in one representative participant. **(A)** |E| on the cortical surface for the isotropic (ISO), DTI, and MD-dMRI models. **(B)** MD-dMRI minus DTI on the same surface, as an absolute difference, and relative to DTI, and the relative difference on a coronal slice through the anode center over a T1 underlay. **(C)** Cumulative distributions of the absolute relative difference over the GM and WM elements of the head mesh, weighted by element volume. A point on a curve gives the share of that tissue whose difference is at or below the value on the *x*-axis. Shading and the dotted line at 10% mark the small and moderate categories. Dotted horizontal lines read the share of each tissue within 10%.

### 3.4 Group comparison (PD vs. HC)

Under the MD-dMRI model, patients and controls were not significantly different in E-field magnitude in any region under any montage (0 of 16 regions per montage, the largest effect in any region and montage was Cohen’s *d* = 0.59, and the smallest uncorrected p was 0.11; age-adjusted Cohen’s *d*, Mann–Whitney on the age residuals; Supplementary Table S3). Every region and montage was equivalent to within 25% of the HC mean by two one-sided tests, a bound at which the comparison had power above 99% at this sample size. At the 10% bound, where power was only 11%, equivalence was established in most regions but not all (Figure 4). Patients and controls were also not significantly different in CSF fraction (Cohen’s *d* = 0.42).

**Figure 4:**
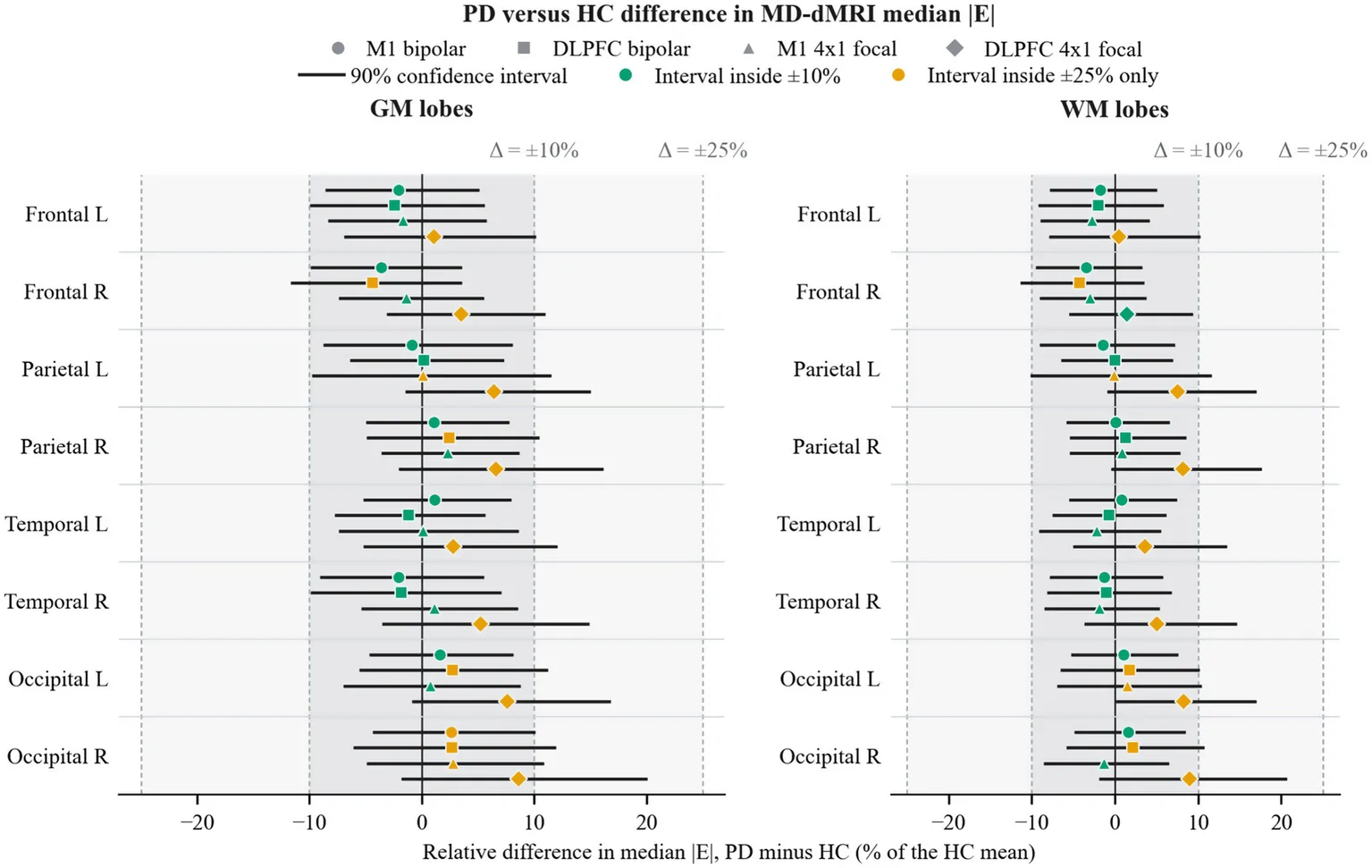
Exploratory comparison of the predicted E-field between patients and controls. Each row is one region, and the four markers within it are the four montages, from top to bottom M1 bipolar (circles), DLPFC bipolar (squares), M1 4 × 1 focal (upward triangles), and DLPFC 4 × 1 focal (diamonds). GM lobes are on the left, and WM lobes are on the right. The marker is the difference between groups in age-adjusted median |E| under the MD-dMRI model, expressed as a percentage of the mean in the HC group. The line is its 90% confidence interval. Shading and the dashed edges mark 10 and 25% of the HC mean. The marker color gives the equivalence verdict from two one-sided tests at alpha 0.05: green if the whole interval lies within 10% and amber if it lies within 25% but not within 10%.

### 3.5 Orientation divergence between the two anisotropic models

The single-shell DTI and QTI mean tensors disagreed mainly in orientation. Across the cohort, their principal directions differed by a median of 18 to 25 degrees across the WM lobes, well above the near-zero angle expected if the two agreed. In GM, the angle rose to about 45 degrees, approaching the 60-degree random-axis chance level without reaching it, where the near-isotropic tensors leave the principal direction ill-defined (Figure 5). A DTI tensor fitted to the QTI acquisition still differed from the mean tensor by 11 to 16 degrees per WM lobe (Figure 5B; Supplementary Table S5). The angle between the two acquisitions was largest at tissue interfaces, where WM meets GM or CSF. The median fell from 24.8 degrees in the WM voxels bordering another tissue to 11.8 degrees in those more than 3 mm away. The orientation difference did not, however, predict a larger E-field difference: Within the WM lobes, participants with a larger V1 angle had a smaller absolute percentage difference in $p_{95}$ |E| between the MD-dMRI and DTI models (*r* = −0.32, 95% subject-clustered bootstrap CI [−0.52, −0.09]). This association attenuated to non-significance once the FA difference between the two tensors was accounted for (partial *r* = −0.06, 95% CI [−0.22, 0.11]), whereas the FA difference still predicted the E-field difference when controlling for the angle (partial *r* = −0.61, [−0.71, −0.47]).

**Figure 5:**
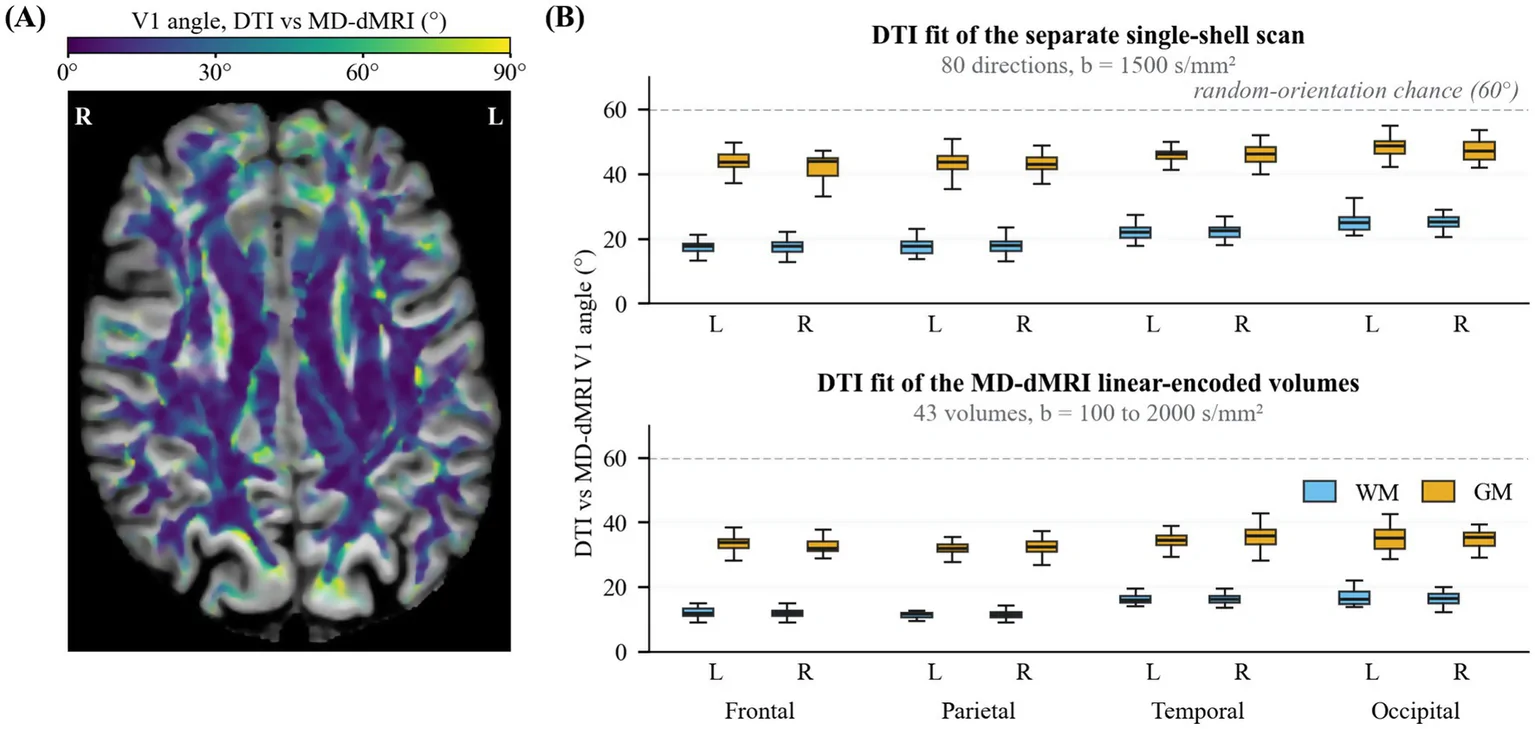
Orientation divergence between the two anisotropic models. Acute angle between the principal diffusion direction (V1) of a DTI tensor and that of the QTI mean tensor. A small angle means the two estimates point nearly the same way. **(A)** Voxel-wise angle, with the DTI tensor fitted to the separate single-shell scan, on a representative transverse slice over a T1 underlay, across the WM and GM mask. Voxel opacity tracks fractional anisotropy, rising from zero at FA = 0.10 to full opacity at FA = 0.30, so strongly anisotropic voxels read solid and nearly isotropic ones fade into the underlay. **(B)** Angles across the cohort: one box per region, WM in blue and GM in orange, left (L) and right (R) hemispheres side by side. Both plots show the angles against the same QTI mean tensor and differ only in which data the DTI tensor was fitted to: the separate single-shell scan, and the linear-encoded volumes of the MD-dMRI scan itself. The dashed line is the chance level for sign-ambiguous directions, the 60-degree median angle between two random three-dimensional axes. Values per region are in Supplementary Table S5.

### 3.6 Association between predicted E-field and MRE stiffness

Whole-brain median ∣E∣ covaried positively with individual MRE stiffness, controlling for group (partial Pearson’s *r* = 0.58, *q* = 0.002; Figure 6A). The correlation was nearly identical across the three conductivity models (ISO, DTI, and MD-dMRI: *r* = 0.579 to 0.592, all *q* = 0.002), so the MD-dMRI model is shown as representative. Further adjusting for age left the association significant (partial *r* = 0.48, *q* = 0.018). Controlling instead for CSF volume attenuated it to non-significance (partial *r* = 0.09, *q* = 0.66). With CSF fraction in place of CSF volume, a sensitivity check, the partial correlation was similarly negligible (*r* = 0.06). Conversely, whole-brain |E| decreased with CSF fraction (*r* = −0.66; Figure 6B). Controlling for stiffness reduced this E-field to CSF correlation only partially (partial *r* = −0.41). Stiffness and CSF fraction were strongly collinear (*r* = −0.84). With both predictors in one multiple regression alongside age and group, CSF fraction remained associated with whole-brain median |E| and stiffness did not remain associated (Table 3, Panel A). Of the variance they explain once those two covariates are accounted for, 3% was unique to stiffness, 42% was unique to CSF fraction, and 55% was common to both (Table 3, Panel B). As an input check, mean diffusivity showed the expected negative correlation with MRE stiffness (Olsson et al., 2025). We do not report it as a finding, since it re-derives their results on the same data.

**Figure 6:**
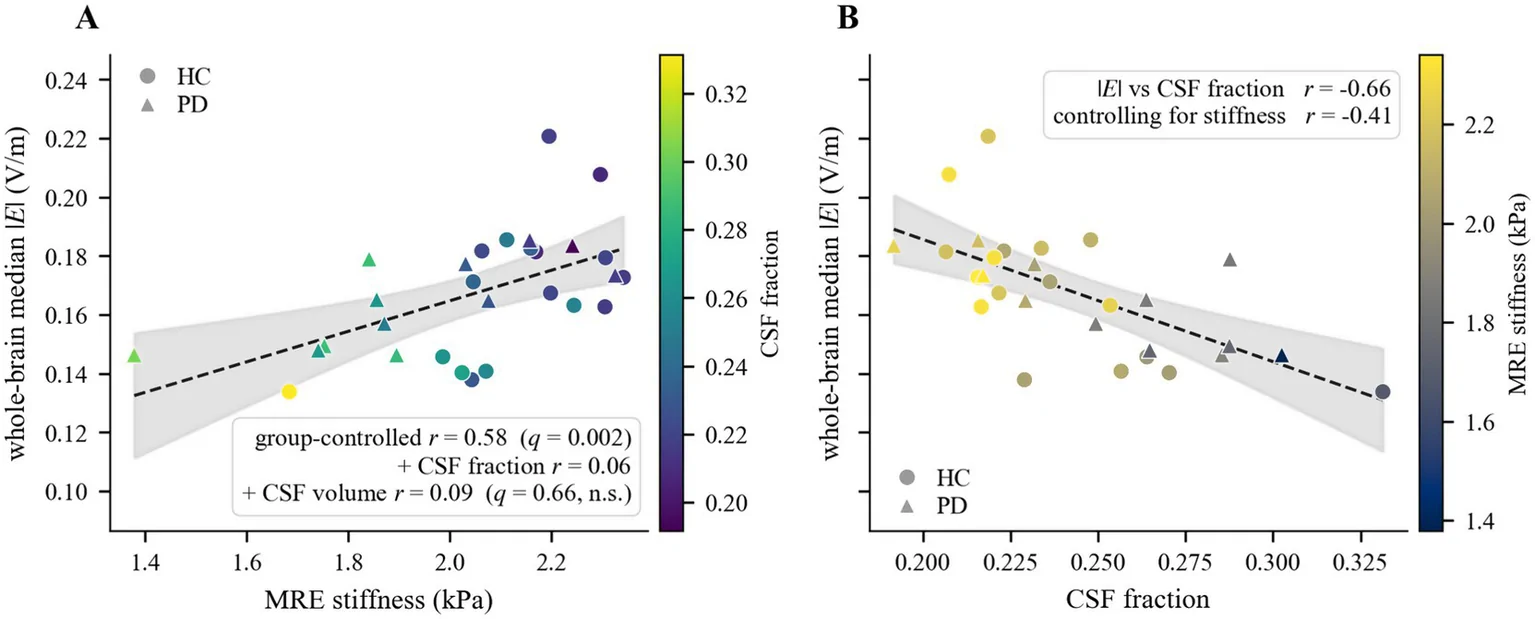
Association between the predicted E-field and MR elastography stiffness. **(A)** Whole-brain median ∣E∣ (MD-dMRI model, M1 bipolar montage) plotted against individual brain stiffness (kPa) for the 29 participants. Markers are colored by CSF fraction and shaped by group (circles, HC; triangles, PD). The dashed line is an ordinary-least-squares fit with its 95% confidence interval. The partial Pearson correlation controlling for group is *r* = 0.58 (*q* = 0.002) and is near-identical across the ISO, DTI, and MD-dMRI models. Controlling for CSF volume reduces the correlation to *r* = 0.09 (*q* = 0.66), and controlling for CSF fraction instead similarly reduces it to *r* = 0.06. **(B)** Whole-brain median ∣E∣ plotted against CSF fraction (*r* = −0.66), colored by stiffness, so each panel’s *x*-axis is the other panel’s color. Controlling for stiffness only reduces the association to *r* = −0.41.

## 4 Discussion

We built, to our knowledge, the first human tDCS conductivity model from the macroscopic mean tensor of MD-dMRI and compared it in a PD cohort against a conventional single-shell DTI tensor and an isotropic model, changing only the conductivity assigned to brain tissue while the head mesh, electrodes, and E-field solver stayed constant. The resulting E-field changed little: It depended on tissue type and was consistent across all four montages, but the change was small and stayed close to both the DTI and the isotropic model. Patients and controls did not differ significantly. The E-field correlated positively with brain stiffness; however, that association attenuated to non-significance once we controlled for CSF fraction, while the E-field’s association with CSF fraction was more robust.

### 4.1 The choice of diffusion tensor is second order for the predicted E-field

Because the multidimensional and single-shell tensors were passed through the same volume-normalized mapping and the same solver, the comparison isolates the effect of the tensor estimate on the E-field. An earlier study instead varied the conductivity mapping formula applied to a single diffusion tensor (Shahid et al., 2013), whereas here the mapping is held constant and only the tensor estimate changes. Such isolation largely preserves orientation, but not magnitude, because the two acquisitions differed in native resolution and smoothing, so the magnitude difference reflects that acquisition mismatch as much as the tensor estimate. This is also distinct from conductivity tensor imaging, which builds conductivity by scaling a conventional diffusion tensor with high-frequency electrical properties rather than from tensor-valued diffusion encoding (Katoch et al., 2023; Sajib et al., 2018; Mandija et al., 2026).

The difference we found was dependent on tissue type and consistent across montages, which lets us attribute it to the model used rather than the electrode montage. GM, which is close to isotropic, showed the three models converging. WM, where the tensor orientations genuinely diverge, showed a consistent E-field difference of 2.7 to 6.6%, although the magnitude of that difference is confounded by acquisition and smoothing (see Section 4.3). This matches what recent work with the same segmentation and mapping tools reported: DTI-based anisotropy adds little to cortical transcranial electrical stimulation predictions and that scalar tissue conductivities are themselves a large source of uncertainty in tDCS modeling (Schmidt et al., 2015; Saturnino et al., 2019b; Mosayebi-Samani et al., 2025). Suh et al. (2012) found that WM anisotropy changed the E-field on the targeted cortical surface by only 1 to 3% but changed the E-field within the WM itself considerably more. In EEG source localization in primary visual cortex, realistic WM anisotropy moved dipole locations by at most 6.2 mm on average (Lee et al., 2009). Published estimates of the size of this effect differ, and they compare anisotropic against isotropic conductivity: GM and WM anisotropy changed the E-field on the cortical middle surface by a root-mean-square of 6.8 to 8.0% relative to the isotropic E-field under the volume-normalized mapping used here (Mosayebi-Samani et al., 2025), and neglecting WM anisotropy changed it by 9.7 to 13.2% in WM, with peak differences in E-field orientation of 23 to 25 degrees (Caiani et al., 2026). Our comparison is between two anisotropic tensors, and by the same root-mean-square metric their E-fields differed by 4.4 to 4.9% over the whole cortical middle surface across the four montages. Because the volume-normalized mapping keeps only the shape and orientation of the tensor, and because the E-field is only modestly sensitive to conductivity anisotropy (Opitz et al., 2011; Suh et al., 2012; Mosayebi-Samani et al., 2025), what separates the two models is orientation (Figure 5). The multidimensional mean tensor is expected to be the less kurtosis-biased, but without a measured conductivity, we cannot show which model is more accurate. What we can say is that changing the tensor changed the predicted E-field only slightly and that the E-field difference did not increase with this orientation difference (Section 3.5). These findings extend to transcranial alternating current stimulation (tACS). In the tACS frequency range, the quasi-static approximation holds. Hence, the E-field is proportional to the instantaneous injected current (Saturnino et al., 2019a), and intracranial recordings during tACS confirmed this linearity in a purely resistive medium (Huang et al., 2017). The differences between conductivity models and the dependence on individual anatomy reported here therefore apply to tACS at the same current amplitude, although its physiological effects are also dependent on stimulation frequency and phase.

### 4.2 Individual anatomy, not the conductivity tensor, governs the predicted E-field

The link between the E-field and brain stiffness is consistent with a morphology effect. The whole-brain correlation was positive and did not depend on group, but it disappeared once we accounted for CSF, whether as total volume or as a fraction of brain plus CSF volume, and it was the same under all three conductivity models. CSF diverts injected current (Huang et al., 2017), a thicker CSF layer lowers the E-field that reached the cortex (Laakso et al., 2015), and its volume is one of the larger sources of between-subject variability in the predicted E-field (Minjoli et al., 2017; Indahlastari et al., 2020). Individual head anatomy, CSF and skull thickness in particular, dominates that variability, to the point where stimulation with a fixed current varies by more than a factor of two across people (Laakso et al., 2015; Evans et al., 2020), and the spread grows with more focal montages such as the 4 × 1 focal arrays used here (Mikkonen et al., 2020). Brain stiffness falls with age, faster than volume does (Sack et al., 2011), and falls further in PD (Lipp et al., 2018; Olsson et al., 2025). Atrophy lowers stiffness and enlarges CSF compartments, which is why stiffness and CSF fraction were strongly collinear in this cohort (Section 3.6). Taking CSF into account eliminated the E-field to stiffness correlation, whereas adjusting for stiffness only partly reduced the E-field to CSF fraction correlation. When both are factored into a joint regression, only CSF fraction retained its association with E-field, and nearly all of the variance explained by stiffness was shared with CSF fraction (Table 3). Since CSF diverts injected current and stiffness has no direct electrical effect on the E-field, this is the expected result. Stiffness appears to track the same morphology that shapes the E-field rather than acting on the E-field through conductivity, which would explain why the association was the same under all three conductivity models.

We found no evidence of a group difference in the E-field in any region or montage. The comparison was underpowered at 29 participants, and we treat it as exploratory, but non-significance is also what the small model effect predicts: If disease changes the diffusion tensor only slightly as far as the E-field is concerned, the E-field will not separate patients from controls. Where predicted E-fields do separate patients from controls, it is generally due to gross macrostructural changes, whether the focal lesions of stroke (Kimura et al., 2026) or the anatomical differences of other clinical cohorts, in which a lower E-field was observed in patients than controls (Mizutani-Tiebel et al., 2022). PD in this cohort is not associated with macrostructural changes of that magnitude, so a group difference would not be expected on anatomical grounds. Thus, we interpret this non-significance as consistent with subtle anatomy. In head models of aging and mild cognitive impairment, atrophy reduced the current density in the brain (Mahdavi and Towhidkhah, 2018), and in cognitively healthy older adults less current reached the brain as atrophy increased (Indahlastari et al., 2020). For diseases characterized by greater atrophy, such as Alzheimer’s disease, current shunting through CSF would be expected to produce larger between-subject differences in the predicted E-field, which is where individualized head modeling has the most to offer. The M1 and DLPFC targets modeled here are those of the first controlled tDCS trial in PD (Fregni et al., 2006). For tDCS in PD, where the therapy shifts cortical excitability in a dose-dependent way with the delivered E-field (Nitsche and Paulus, 2000; Jamil et al., 2017) yet stays clinically modest and variable (Benninger et al., 2010; Broeder et al., 2015; Lefaucheur et al., 2017; Elsner et al., 2016), the individualization that matters is anatomical. A standard single-shell diffusion tensor already captures the anisotropy to which the predicted E-field is sensitive, so modeling efforts are better spent on accurate CSF and atrophy segmentation, or on E-field-based dose normalization (Evans et al., 2020), rather than on refining the diffusion tensor.

### 4.3 Limitations

This study has several limitations. The two anisotropic models came from separate diffusion scans that differed in resolution and smoothing, so the WM part of the magnitude difference is partly a preprocessing artifact rather than a property of the tensors. The smoothing that stabilizes the covariance fit also lowers its WM anisotropy, and removing it does not fix the comparison, because the unsmoothed fit is inflated by noise and non-positive-definite in a median of 11% of voxels. The WM difference in p_95_ |E| increased in magnitude with the smoothing kernel: Across the eight WM lobes, it is −1.5 to 0.3% without smoothing, −10.2 to −5.3% at 1.4 voxels, and −1.3 to 0.0% when the DTI tensor is instead smoothed to the effective resolution of the MD-dMRI tensor (Supplementary Table S2). In GM, where the MD-dMRI E-field sits above DTI (Section 3.2), the difference follows the same pattern: It increases with the kernel and is negative in every GM lobe under that matched smoothing. Because the two scans were separate, each tensor was aligned to the head model by its own 12-DOF affine. This does not bias the within-subject E-field comparison, where each tensor is placed correctly in the subject’s own anatomy. Fitting a DTI tensor to the QTI acquisition itself (Section 3.5) shows that approximately two thirds of the orientation divergence persists when scan, resolution, preprocessing, and smoothing are identical and no registration enters the comparison, so it is largely a property of the estimation model rather than of the acquisition. It may, however, partly reflect angular sampling. The DTI acquisition used 80 diffusion directions, whereas the linear encoding of the MD-dMRI acquisition sampled 6 to 21 directions per shell. The mean tensor is therefore expected to be less kurtosis-biased but more affected by the lower angular resolution of its acquisition, and we interpret the orientation divergence as a difference between the tensor estimates rather than as evidence that either orientation is more accurate. At tissue interfaces, where partial volume effects are expected, the orientation divergence was approximately twice that in WM which is more than 3 mm from them (Section 3.5), so the regional angles overstate the divergence away from the interfaces, which is still about 12 degrees. The cohort is small and predominantly male (see Olsson et al., 2025), which limits both the power of the group comparison and sex-stratified generalizability. With 12 patients and 17 controls, the comparison could detect only large effects (Section 2.8), and approximately 26, 64, and 176 participants per group would be needed for effects of *d* = 0.8, 0.5, and 0.3. Establishing equivalence within 10% at 80% power would need approximately 38 per group, against a power of 11% at the present size. The 10 and 25% equivalence bounds describe the accuracy an E-field simulation needs for reliable dose quantification rather than a threshold for clinical effect (Kimura et al., 2026). The stiffness association is cross-sectional, and its covariates (CSF volume, age, and stiffness) are collinear, so even though the E-field’s association is with CSF rather than stiffness, this is still consistent with a shared morphology driver, not proof of a mediation pathway. The study is computational, with no measured E-field to validate against. Intracranial recordings are themselves too noisy to adjudicate modeling choices such as anisotropy (Puonti et al., 2019), but non-invasive magnetic resonance current density imaging provides a forward route (Göksu et al., 2018). Despite these limitations, the within-subject comparison is internally stable. The WM lobe difference is consistent in sign in every participant under the M1 bipolar montage, so it does not depend on how we corrected for multiple comparisons. The tensor-estimate claim rests on the orientation divergence, and the agreement between the three models on E-field magnitude and the collapse of the stiffness association under CSF control are further checks that do not favor any single modeling choice.

### 4.4 Future directions

Matching the two diffusion acquisitions in resolution and smoothing would separate the tensor effect from preprocessing and let the magnitude difference be read directly. A single macroscopic tensor cannot separate microscopic anisotropy from orientation dispersion, so it understates conductivity anisotropy where fibers cross or disperse. MD-dMRI separates the two and, through the covariance of the intra-voxel tensor distribution, resolves microstructure that a single mean tensor sets aside (Westin et al., 2016; Topgaard, 2017; Lampinen et al., 2017). Building a conductivity model on this richer microstructure is a natural next step, although because these microscopic measures have no orientation of their own (Westin et al., 2016), such a model would have to combine them with a fiber orientation distribution rather than read a conductivity tensor from them directly, and it would need an acquisition able to estimate the intra-voxel diffusion variance reliably (Szczepankiewicz et al., 2019). Combined with direct validation against a measured conductivity, such a model could reflect the electrical properties of WM more accurately than a single macroscopic tensor allows.

## 5 Conclusion

We showed that a tDCS conductivity model can be built from MD-dMRI and that its predictions stay close to those of the DTI and isotropic models in PD. The model choice shifted the predicted E-field in a tissue-dependent way, but the shift between the two anisotropic models was at most 6.6% of the DTI in any region or montage, averaged across the cohort. Because the volume-normalized mapping scales every tensor to the same volume before the tissue conductivity is applied, the two anisotropic models can differ in the predicted E-field only through tensor shape and orientation, and the shape difference itself is confounded by the resolution and smoothing of the two acquisitions. What we can attribute to the tensor estimate is therefore a difference in orientation. Patients and controls showed no detectable difference in this underpowered, exploratory comparison. For tDCS dosimetry in PD, then, it is individual anatomy, more than the choice of diffusion tensor, that governs the predicted E-field, so individualized dosing is better served by CSF- and atrophy-aware head modeling than by a more elaborate diffusion tensor. The value of MD-dMRI may ultimately lie less in the mean tensor than in the microstructure it also resolves. Whether that can yield a more accurate conductivity model is an open question, and one that would need to be validated with measured conductivity.

## Data Availability

The MR imaging data analyzed here are subject to ethical and legal restrictions and cannot be shared publicly. Anonymized data can be made available to researchers who meet the applicable legal and ethical requirements, on reasonable request to the corresponding author. The analysis and modeling code is openly available at https://github.com/santioso-hue/MRE-tDCS-PD.
