# Supplementary material for: Anisotropic conductivity modeling for tDCS in Parkinson’s disease using multidimensional diffusion MRI
Source: Front Hum Neurosci. 2026 Oct 21;20:1972676. doi: 10.3389/fnhum.2026.1972676 (PMC13730951; doi:10.3389/fnhum.2026.1972676)
Supplement: Supplementary file 1 [file Table_1.DOCX]

# Supplementary material

**S-Table 1.** Diffusion MRI preprocessing, tensor fitting, registration, conductivity mapping and simulation parameters (Panel A), and the thresholds at which the automated quality control checks flag a participant for inspection (Panel B). Section 1 of Panel A was performed by the cohort pipeline that supplied the corrected data.

| Step | Software (version) | Setting |
| --- | --- | --- |
| **Panel A. Pipeline parameters** | | |
| **1. Upstream preprocessing** | | |
| 1.1 Susceptibility distortion correction, single-shell DTI scan | FSL topup (6.0.7.22 or earlier) | phase encoding = A-P / P-A; readout = 0.052 s; warpres = 20 to 4 mm over 9 levels; subsamp = 2, 1; fwhm = 8 to 0 mm; miter = 5 to 20; regmod = bending energy; splineorder = 3; interp = spline; scale = on. FSL b02b0 configuration. |
| 1.2 Eddy current and motion correction, single-shell DTI scan | FSL eddy_openmp (6.0.7.22 or earlier) | required inputs only, no optional flags (--imain, --mask, --acqp, --index, --bvecs, --bvals, --topup); all modeling therefore at eddy defaults: flm = quadratic; slm = none; mporder = 0, volume to volume; niter = 5; interp = spline; resamp = jac; repol = off; peas = on. Mask from the topup-corrected b = 0. 81 volumes, 80 at b = 1500 s/mm² and one at b = 0; gradients rotated by eddy. |
| 1.3 Synthetic undistorted b = 0, tensor-valued scan | Synb0-DisCo | no user-set options; one synthetic undistorted b = 0 predicted from the distorted b = 0 and the T1. |
| 1.4 Susceptibility distortion correction, tensor-valued scan | FSL topup | warpres = 20 to 4 mm over 7 levels; subsamp = 2, 1; fwhm = 8 to 1 mm; miter = 5 to 15; regmod = bending energy; splineorder = 3; interp = spline; scale = on. Synb0-DisCo configuration. |
| 1.5 Eddy current and motion correction, tensor-valued scan | FSL eddy (6.0.7.22 or earlier) | topup field from step 1.4; mask from FSL bet on the b = 0. Linear encoding 44 volumes, spherical encoding 38; b = 0, 100, 700, 1400, 2000 s/mm²; 2.5 mm isotropic. |
| **2. Head model and parcellation** | | |
| 2.1 Head model and mesh | SimNIBS charm 4.6.0 | --forceqform, the only argument away from the defaults; inputs the FreeSurfer conformed T1 and T2, qform and sform stamped, so the two segmentations share one grid. Single-threaded (OMP and ITK threads 1). Mesh: median 4.15 M tetrahedra, range 3.78 to 4.88 M; median 746 k nodes, range 684 to 875 k. |
| 2.2 GM and WM lobe labels | FreeSurfer 7.2.0 | recon-all output consumed, not re-run here. GM lobes from the Desikan-Killiany labels of aparc+aseg, 1000 + index left and 2000 + index right; WM lobes from wmparc, 3000 + index left and 4000 + index right. Cingulate folded anterior into frontal and posterior into parietal; insula excluded. |
| 2.3 Lobe labels into head model space | FSL FLIRT 6.0.7.22 | recon-all orig to charm T1: dof = 6; cost = mutualinfo; search = ±20 degrees about each axis. Labels interp = nearestneighbour. |
| 2.4 Restriction of each region to its tissue | Python 3.11.14 | region labels restricted to their own tissue: GM lobes to charm tissue tag 2, WM lobes to tag 1. |
| **3. QTI covariance fit** | | |
| 3.1 Co-registration of the two encoding series | elastix 5.3.1 | affine, elastix_p_affine(100); reference volumes b at or below 1100 s/mm². |
| 3.2 Smoothing before the covariance fit | md-dmri framework | isotropic Gaussian; filter_sigma = 0.7 voxels, that is 1.75 mm on the 2.5 mm grid. Sensitivity refits at 0, 0.4, 0.5 and 1.4 voxels. |
| 3.3 Brain mask for the covariance fit | md-dmri framework | mdm_s_mask with mio_mask_threshold; threshold = 0.1 of the 99th percentile of the b = 0 image, the toolbox default. |
| 3.4 QTI covariance fit, mean tensor | md-dmri framework | dtd_covariance_opt(mdm_opt()), two fields overridden: do_overwrite = 1; filter_sigma = 0.7. All other options default. b_delta = 1 linear, 0 spherical; 82 merged volumes. Mean tensor from cov_mfs.m(:,:,:,2:7), Mandel Voigt order. |
| **4. Mean tensor conditioning** | | |
| 4.1 Degenerate mean tensor substitution | Python 3.11.14 | all three eigenvalues set to the voxel mean diffusivity, floored at 0.001 µm²/ms, when the smallest eigenvalue <= 0.001 µm²/ms or the mean diffusivity < 0.2 µm²/ms. |
| **5. Registration and tensor reorientation** | | |
| 5.1 Diffusion to T1 registration, MD-dMRI | FSL FLIRT 6.0.7.22 | model S0 to the brain-masked charm T2. Stage 1: dof = 6; cost = mutualinfo; search = ±25 degrees about each axis. Stage 2: dof = 12; cost = mutualinfo; initialized from stage 1. Affine rejected when \|det\| of its linear part is outside 0.3 to 3. |
| 5.2 Tensor reorientation | FSL vecreg 6.0.7.22 | principal eigenvector and triaxial tensor: reoriented with the affine. Eigenvalues: interp = trilinear as independent scalar maps, then re-sorted. Diffusion mask: interp = nearestneighbour. |
| **6. Single-shell DTI tensor** | | |
| 6.1 Single-shell tensor fit | FSL dtifit 6.0.7.22 | ordinary least squares, the program default, no -w weighting flag; --save_tensor; -m the topup-corrected b = 0 brain mask delivered with the scan. Single shell b = 1500 s/mm². Fallback only when the delivered b-values are missing or mismatched: b = 0 taken from the zero-norm column of the gradient table. |
| 6.2 DTI tensor into the head model | SimNIBS dwi2cond 4.6.0 | --all --regmthd=12dof, the only non-default setting (the default is nonlinear FNIRT); FA registered to T1 by a 12 dof FLIRT affine. dwi2cond preprocessing not re-run on already corrected data. |
| 6.3 Control fit, DTI of the MD-dMRI data (not part of the per-subject pipeline) | FSL dtifit 6.0.7.22 | ordinary least squares, no -w flag. 43 diffusion-weighted linear-encoded volumes for the main fit, the linear series of step 1.5 without its b = 0 volume, and 23 volumes for the b = 2000 s/mm² sensitivity fit, that shell with its b = 0 volumes, both from the motion-corrected and smoothed series the covariance fit consumed. |
| **7. Conductivity mapping** | | |
| 7.1 Diffusion to conductivity rule | SimNIBS 4.6.0 | anisotropy_type vn: sigma = sigma0 D / det(D)^(1/3), retaining only the shape and orientation of D. sigma0, the literature isotropic tissue conductivity: 0.126 S/m WM; 0.275 S/m GM. Identical for the DTI and MD-dMRI tensors. |
| 7.2 Conductivity tensor caps | SimNIBS 4.6.0 | aniso_maxratio = 10, eigenvalue ratio capped at 10 to 1; aniso_maxcond = 2, capping the geometric-mean-normalized eigenvalue, an effective ceiling of 2 sigma0 and the binding cap. Both are SimNIBS defaults, identical for both anisotropic models. |
| **8. Simulation** | | |
| 8.1 Electrodes and injected current | SimNIBS 4.6.0 | bipolar montages: two rectangular pads, 5 x 5 cm, 4 mm thick, +2 and -2 mA. 4 x 1 focal montages: five disc electrodes, 12 mm diameter, 4 mm thick; +2 mA at the center and -0.5 mA at each of the four surrounding electrodes. |
| 8.2 FEM solver | SimNIBS 4.6.0 with PETSc | SimNIBS default solver, not overridden: conjugate gradient; HYPRE BoomerAMG preconditioner; HMIS coarsening; relative tolerance 1e-10. E-field magnitude and vector both written. |

**Panel B. Quality control**

| Check | Definition | Flagged when |
| --- | --- | --- |
| Mean tensor degeneracy | fraction of the mask given the isotropic substitution of step 4.1 | > 0.25 |
| Diffusion field of view containment | fraction of the head model brain labels inside the registered diffusion mask | < 0.95 |
| Edge alignment, diffusion to T1 | Pearson r of the gradient magnitudes of the registered S0 and the T1, brain eroded by 2 voxels, Gaussian sigma 1 voxel | < 0.08 |
| Reorientation, corpus callosum | median absolute left-right component of the unit principal eigenvector in the corpus callosum | < 0.6 |
| Reorientation, cerebral peduncle | median absolute superior-inferior component of the principal eigenvector in the midbrain, after the SimNIBS pre-FEM rotation | < 0.55 |
| Reorientation cross-check | fraction of WM voxels where the reoriented principal eigenvector and the independently reoriented tensor axis agree within 15 degrees | < 0.90; < 0.70 for the 0, 0.4 and 0.5 voxel refits |
| dwi2cond tensor | mean FA in the corpus callosum; mean FA in cerebrospinal fluid; fraction of voxels with a non-positive smallest eigenvalue | < 0.25; > 0.25; > 0.001 |
| Simulated E-field range | 95th percentile of \|E\| over the GM elements at 2 mA, each conductivity model | outside 0.10 to 0.60 V/m |
| Simulation spike | ratio of the maximum to the 95th percentile of \|E\| over the GM elements, each conductivity model | > 8 |
| Mesh quality | median tetrahedron quality; fraction of tetrahedra below 0.10 | < 0.10; > 0.05 |
| Cohort outliers | deviation from the cohort median, in median absolute deviations | > 3, applied once four or more participants are processed |

**S-Table 2.** Sensitivity of the MD-dMRI versus DTI comparison to the Gaussian smoothing applied to the QTI signal before the covariance fit. Each row is one kernel width; the last row instead smooths the DTI tensor to the effective resolution of the 0.7 voxel fit. Non-positive-definite voxels are a percentage of the diffusion brain mask, cohort median with the cohort maximum in parentheses. The E-field columns are MD-dMRI minus DTI as a percentage of DTI, under the M1 bipolar montage. Every other cell is the range of the per-region cohort median across the eight WM lobes, or the eight GM lobes in the GM column.

| Kernel (voxels) | Non-positive-definite voxels (%) | p95 \|E\| difference, WM (%) | p95 \|E\| difference, GM (%) | V1 angle, WM (degrees) | Mean tensor FA | FA difference, mean tensor minus DTI |
| --- | --- | --- | --- | --- | --- | --- |
| 0 | 11.1 (15.9) | -1.5 to 0.3 | 0.1 to 0.8 | 19.9 to 26.7 | 0.320 to 0.380 | 0.052 to 0.105 |
| 0.4 | 8.3 (12.0) | -2.3 to -0.9 | 0.0 to 0.9 | 19.2 to 26.1 | 0.282 to 0.341 | 0.020 to 0.068 |
| 0.5 | 4.9 (8.1) | -3.7 to -2.2 | -0.1 to 1.2 | 18.2 to 25.2 | 0.230 to 0.284 | -0.025 to 0.015 |
| 0.7 (used in this study) | 1.5 (4.6) | -6.3 to -3.6 | -0.2 to 1.4 | 17.6 to 25.2 | 0.173 to 0.219 | -0.083 to -0.052 |
| 1.4 | 0.05 (0.41) | -10.2 to -5.3 | -0.2 to 1.9 | 20.2 to 31.7 | 0.098 to 0.134 | -0.177 to -0.125 |
| 0.7, with the DTI tensor smoothed to the same effective resolution | not applicable | -1.3 to 0.0 | -0.7 to -0.2 | 12.9 to 20.7 | 0.173 to 0.219 | 0.007 to 0.033 |

**S-Table 3.** Region-wise paired comparison of the predicted E-field, MD-dMRI versus DTI, for the 16 regions and the whole brain under each of the four montages, with the exploratory patient versus control comparison. ISO, MD-dMRI and DTI are the cohort median of the within-subject 95th percentile E-field magnitude; the remaining model comparison columns are as in Table 2. q is the Benjamini-Hochberg adjustment of the uncorrected Wilcoxon signed-rank p across the 16 regions within each montage, so the whole brain row carries none. d is the age-adjusted Cohen's d of the median E-field magnitude, PD minus HC, with its uncorrected Mann-Whitney p.

| Montage | Region | ISO (V/m) | MD-dMRI (V/m) | DTI (V/m) | MD-dMRI minus DTI (V/m) | 95% CI (V/m) | r_rb_ | Relative difference, mean [SD] (%) | Relative difference, min to max (%) | p | q | PD vs HC d | PD vs HC p |
| --- | --- | --- | --- | --- | --- | --- | --- | --- | --- | --- | --- | --- | --- |
| M1 bipolar | Frontal GM L | 0.365 | 0.367 | 0.366 | 0.0011 | [0.0007, 0.0030] | 0.694 | 0.62 [1.02] | -0.89 to 4.22 | 0.001 | 0.001 | -0.15 | 0.744 |
| M1 bipolar | Frontal GM R | 0.353 | 0.365 | 0.347 | 0.0028 | [0.0018, 0.0041] | 0.862 | 0.94 [1.27] | -0.58 to 6.71 | <0.001 | <0.001 | -0.27 | 0.419 |
| M1 bipolar | Parietal GM L | 0.300 | 0.297 | 0.294 | 0.0042 | [0.0027, 0.0050] | 1.000 | 1.33 [0.68] | 0.03 to 3.01 | <0.001 | <0.001 | -0.06 | 0.913 |
| M1 bipolar | Parietal GM R | 0.189 | 0.184 | 0.184 | 0.0005 | [0.0002, 0.0018] | 0.476 | 0.51 [1.22] | -2.10 to 4.32 | 0.024 | 0.028 | 0.10 | 0.744 |
| M1 bipolar | Temporal GM L | 0.255 | 0.259 | 0.259 | 0.0012 | [0.0001, 0.0017] | 0.584 | 0.44 [0.75] | -0.90 to 2.52 | 0.006 | 0.007 | 0.10 | 0.711 |
| M1 bipolar | Temporal GM R | 0.199 | 0.195 | 0.197 | -0.0005 | [-0.0013, 0.0003] | -0.191 | -0.12 [1.02] | -2.11 to 2.56 | 0.381 | 0.381 | -0.16 | 0.647 |
| M1 bipolar | Occipital GM L | 0.177 | 0.179 | 0.176 | 0.0013 | [0.0003, 0.0021] | 0.779 | 0.83 [1.00] | -0.92 to 3.10 | <0.001 | <0.001 | 0.14 | 0.419 |
| M1 bipolar | Occipital GM R | 0.139 | 0.137 | 0.135 | 0.0001 | [-0.0002, 0.0009] | 0.283 | 0.38 [1.35] | -2.06 to 5.04 | 0.190 | 0.203 | 0.21 | 0.347 |
| M1 bipolar | Frontal WM L | 0.514 | 0.536 | 0.568 | -0.0258 | [-0.0340, -0.0226] | -1.000 | -5.20 [1.63] | -9.09 to -1.77 | <0.001 | <0.001 | -0.14 | 0.777 |
| M1 bipolar | Frontal WM R | 0.503 | 0.528 | 0.542 | -0.0246 | [-0.0297, -0.0191] | -1.000 | -4.63 [1.43] | -8.30 to -2.19 | <0.001 | <0.001 | -0.27 | 0.471 |
| M1 bipolar | Parietal WM L | 0.433 | 0.459 | 0.476 | -0.0221 | [-0.0281, -0.0194] | -1.000 | -5.16 [1.85] | -8.33 to -0.96 | <0.001 | <0.001 | -0.10 | 0.777 |
| M1 bipolar | Parietal WM R | 0.269 | 0.286 | 0.294 | -0.0121 | [-0.0146, -0.0106] | -1.000 | -4.33 [1.39] | -6.96 to -1.96 | <0.001 | <0.001 | 0.00 | 0.679 |
| M1 bipolar | Temporal WM L | 0.362 | 0.395 | 0.423 | -0.0262 | [-0.0316, -0.0238] | -1.000 | -6.40 [1.68] | -9.13 to -3.31 | <0.001 | <0.001 | 0.06 | 0.527 |
| M1 bipolar | Temporal WM R | 0.239 | 0.244 | 0.252 | -0.0098 | [-0.0109, -0.0074] | -1.000 | -3.62 [1.42] | -6.22 to -0.23 | <0.001 | <0.001 | -0.11 | 0.744 |
| M1 bipolar | Occipital WM L | 0.251 | 0.273 | 0.285 | -0.0155 | [-0.0172, -0.0121] | -1.000 | -5.17 [1.41] | -7.67 to -2.60 | <0.001 | <0.001 | 0.09 | 0.347 |
| M1 bipolar | Occipital WM R | 0.177 | 0.183 | 0.190 | -0.0076 | [-0.0096, -0.0069] | -1.000 | -4.35 [1.44] | -6.82 to -1.70 | <0.001 | <0.001 | 0.14 | 0.499 |
| M1 bipolar | Whole brain | 0.365 | 0.373 | 0.386 | -0.0077 | [-0.0092, -0.0064] | -1.000 | -2.19 [0.84] | -3.95 to -0.43 | <0.001 | n/a | -0.07 | >0.999 |
| DLPFC bipolar | Frontal GM L | 0.304 | 0.305 | 0.296 | 0.0050 | [0.0044, 0.0056] | 1.000 | 1.73 [0.68] | 0.31 to 3.22 | <0.001 | <0.001 | -0.16 | 0.913 |
| DLPFC bipolar | Frontal GM R | 0.331 | 0.343 | 0.328 | 0.0057 | [0.0041, 0.0075] | 1.000 | 1.81 [1.05] | 0.06 to 4.94 | <0.001 | <0.001 | -0.30 | 0.556 |
| DLPFC bipolar | Parietal GM L | 0.119 | 0.119 | 0.118 | 0.0007 | [0.0000, 0.0015] | 0.517 | 0.57 [1.35] | -3.38 to 4.00 | 0.014 | 0.015 | 0.02 | 0.744 |
| DLPFC bipolar | Parietal GM R | 0.097 | 0.097 | 0.096 | 0.0007 | [0.0005, 0.0008] | 0.864 | 0.79 [0.81] | -1.44 to 2.55 | <0.001 | <0.001 | 0.18 | 0.419 |
| DLPFC bipolar | Temporal GM L | 0.138 | 0.140 | 0.138 | 0.0013 | [0.0012, 0.0023] | 0.913 | 1.13 [0.93] | -1.40 to 3.53 | <0.001 | <0.001 | -0.09 | 0.777 |
| DLPFC bipolar | Temporal GM R | 0.142 | 0.142 | 0.141 | 0.0003 | [-0.0006, 0.0010] | 0.209 | 0.22 [1.05] | -1.78 to 2.62 | 0.325 | 0.325 | -0.13 | 0.777 |
| DLPFC bipolar | Occipital GM L | 0.074 | 0.074 | 0.073 | 0.0007 | [0.0006, 0.0013] | 0.968 | 1.29 [0.96] | -0.57 to 3.32 | <0.001 | <0.001 | 0.19 | 0.211 |
| DLPFC bipolar | Occipital GM R | 0.063 | 0.064 | 0.063 | 0.0010 | [0.0007, 0.0012] | 0.890 | 1.43 [1.26] | -1.30 to 5.37 | <0.001 | <0.001 | 0.17 | 0.263 |
| DLPFC bipolar | Frontal WM L | 0.445 | 0.470 | 0.497 | -0.0254 | [-0.0308, -0.0224] | -1.000 | -5.61 [1.65] | -9.39 to -2.57 | <0.001 | <0.001 | -0.14 | >0.999 |
| DLPFC bipolar | Frontal WM R | 0.495 | 0.514 | 0.536 | -0.0239 | [-0.0282, -0.0207] | -1.000 | -4.79 [1.32] | -7.40 to -2.21 | <0.001 | <0.001 | -0.29 | 0.616 |
| DLPFC bipolar | Parietal WM L | 0.170 | 0.183 | 0.186 | -0.0092 | [-0.0106, -0.0072] | -1.000 | -4.71 [1.78] | -7.28 to -1.53 | <0.001 | <0.001 | 0.00 | 0.744 |
| DLPFC bipolar | Parietal WM R | 0.143 | 0.151 | 0.161 | -0.0067 | [-0.0082, -0.0054] | -1.000 | -4.19 [1.56] | -7.45 to -1.50 | <0.001 | <0.001 | 0.10 | 0.527 |
| DLPFC bipolar | Temporal WM L | 0.201 | 0.224 | 0.235 | -0.0097 | [-0.0125, -0.0084] | -1.000 | -4.45 [1.67] | -7.53 to -1.27 | <0.001 | <0.001 | -0.06 | 0.586 |
| DLPFC bipolar | Temporal WM R | 0.181 | 0.188 | 0.194 | -0.0063 | [-0.0077, -0.0059] | -1.000 | -3.50 [1.31] | -6.68 to -0.84 | <0.001 | <0.001 | -0.08 | 0.879 |
| DLPFC bipolar | Occipital WM L | 0.112 | 0.120 | 0.125 | -0.0057 | [-0.0071, -0.0052] | -0.995 | -4.59 [1.82] | -7.20 to 0.24 | <0.001 | <0.001 | 0.12 | 0.347 |
| DLPFC bipolar | Occipital WM R | 0.088 | 0.095 | 0.098 | -0.0046 | [-0.0058, -0.0038] | -1.000 | -4.72 [1.37] | -7.30 to -1.01 | <0.001 | <0.001 | 0.15 | 0.419 |
| DLPFC bipolar | Whole brain | 0.281 | 0.290 | 0.293 | -0.0022 | [-0.0029, -0.0015] | -0.986 | -0.84 [0.59] | -2.57 to 0.20 | <0.001 | n/a | -0.04 | 0.811 |
| M1 4 x 1 focal | Frontal GM L | 0.242 | 0.243 | 0.240 | 0.0021 | [0.0015, 0.0036] | 0.963 | 1.14 [0.88] | -0.47 to 4.02 | <0.001 | <0.001 | -0.13 | 0.913 |
| M1 4 x 1 focal | Frontal GM R | 0.061 | 0.060 | 0.057 | 0.0022 | [0.0018, 0.0024] | 1.000 | 3.87 [1.23] | 1.57 to 5.89 | <0.001 | <0.001 | -0.12 | 0.879 |
| M1 4 x 1 focal | Parietal GM L | 0.256 | 0.257 | 0.258 | 0.0013 | [0.0009, 0.0020] | 0.844 | 0.52 [0.54] | -1.38 to 1.38 | <0.001 | <0.001 | 0.01 | 0.879 |
| M1 4 x 1 focal | Parietal GM R | 0.056 | 0.058 | 0.056 | 0.0010 | [0.0007, 0.0012] | 0.972 | 1.74 [1.05] | -1.00 to 3.99 | <0.001 | <0.001 | 0.21 | 0.325 |
| M1 4 x 1 focal | Temporal GM L | 0.162 | 0.167 | 0.164 | 0.0025 | [0.0013, 0.0029] | 1.000 | 1.45 [0.88] | 0.01 to 3.16 | <0.001 | <0.001 | 0.01 | 0.879 |
| M1 4 x 1 focal | Temporal GM R | 0.023 | 0.023 | 0.022 | 0.0004 | [0.0002, 0.0004] | 0.949 | 1.57 [1.16] | -0.32 to 3.82 | <0.001 | <0.001 | 0.10 | 0.948 |
| M1 4 x 1 focal | Occipital GM L | 0.054 | 0.056 | 0.055 | 0.0011 | [0.0010, 0.0014] | 0.995 | 2.00 [0.97] | -0.02 to 3.93 | <0.001 | <0.001 | 0.05 | 0.948 |
| M1 4 x 1 focal | Occipital GM R | 0.030 | 0.029 | 0.029 | 0.0003 | [0.0001, 0.0004] | 0.793 | 0.77 [0.87] | -0.91 to 2.39 | <0.001 | <0.001 | 0.20 | 0.347 |
| M1 4 x 1 focal | Frontal WM L | 0.311 | 0.329 | 0.340 | -0.0158 | [-0.0174, -0.0121] | -1.000 | -4.77 [1.70] | -8.66 to -2.61 | <0.001 | <0.001 | -0.24 | 0.471 |
| M1 4 x 1 focal | Frontal WM R | 0.092 | 0.100 | 0.103 | -0.0031 | [-0.0038, -0.0025] | -1.000 | -3.27 [1.13] | -5.43 to -1.14 | <0.001 | <0.001 | -0.26 | 0.711 |
| M1 4 x 1 focal | Parietal WM L | 0.339 | 0.353 | 0.363 | -0.0152 | [-0.0182, -0.0128] | -1.000 | -4.58 [1.65] | -7.74 to -1.85 | <0.001 | <0.001 | 0.00 | 0.948 |
| M1 4 x 1 focal | Parietal WM R | 0.082 | 0.088 | 0.090 | -0.0027 | [-0.0034, -0.0024] | -0.977 | -3.34 [1.98] | -6.69 to 1.47 | <0.001 | <0.001 | 0.07 | 0.499 |
| M1 4 x 1 focal | Temporal WM L | 0.222 | 0.237 | 0.260 | -0.0165 | [-0.0188, -0.0133] | -1.000 | -6.47 [1.82] | -9.18 to -3.21 | <0.001 | <0.001 | -0.17 | 0.616 |
| M1 4 x 1 focal | Temporal WM R | 0.034 | 0.035 | 0.037 | -0.0016 | [-0.0018, -0.0016] | -1.000 | -4.39 [1.29] | -8.34 to -1.26 | <0.001 | <0.001 | -0.15 | 0.616 |
| M1 4 x 1 focal | Occipital WM L | 0.085 | 0.087 | 0.091 | -0.0038 | [-0.0047, -0.0031] | -0.986 | -3.99 [2.02] | -8.87 to 0.52 | <0.001 | <0.001 | 0.10 | 0.303 |
| M1 4 x 1 focal | Occipital WM R | 0.038 | 0.040 | 0.041 | -0.0011 | [-0.0013, -0.0008] | -0.982 | -2.73 [1.54] | -5.31 to 0.49 | <0.001 | <0.001 | -0.10 | 0.845 |
| M1 4 x 1 focal | Whole brain | 0.204 | 0.206 | 0.207 | -0.0011 | [-0.0015, -0.0006] | -1.000 | -0.56 [0.36] | -1.23 to -0.01 | <0.001 | n/a | -0.01 | 0.711 |
| DLPFC 4 x 1 focal | Frontal GM L | 0.245 | 0.244 | 0.245 | 0.0012 | [0.0005, 0.0017] | 0.743 | 0.49 [0.80] | -2.34 to 1.71 | <0.001 | <0.001 | 0.07 | 0.948 |
| DLPFC 4 x 1 focal | Frontal GM R | 0.073 | 0.072 | 0.072 | 0.0007 | [0.0005, 0.0010] | 0.857 | 1.13 [1.12] | -1.46 to 3.48 | <0.001 | <0.001 | 0.27 | 0.527 |
| DLPFC 4 x 1 focal | Parietal GM L | 0.080 | 0.081 | 0.081 | 0.0003 | [-0.0003, 0.0010] | 0.278 | 0.51 [1.67] | -2.54 to 3.64 | 0.191 | 0.204 | 0.47 | 0.166 |
| DLPFC 4 x 1 focal | Parietal GM R | 0.030 | 0.030 | 0.030 | -0.0005 | [-0.0006, -0.0004] | -0.968 | -1.46 [0.90] | -2.92 to 0.75 | <0.001 | <0.001 | 0.43 | 0.211 |
| DLPFC 4 x 1 focal | Temporal GM L | 0.074 | 0.074 | 0.074 | 0.0007 | [0.0001, 0.0013] | 0.637 | 1.03 [1.51] | -1.59 to 5.39 | 0.002 | 0.002 | 0.19 | 0.616 |
| DLPFC 4 x 1 focal | Temporal GM R | 0.027 | 0.028 | 0.027 | 0.0001 | [0.0000, 0.0003] | 0.695 | 0.88 [1.45] | -0.53 to 5.47 | 0.001 | 0.002 | 0.33 | 0.444 |
| DLPFC 4 x 1 focal | Occipital GM L | 0.019 | 0.019 | 0.019 | -0.0001 | [-0.0002, 0.0001] | -0.168 | 0.05 [1.92] | -2.07 to 5.31 | 0.430 | 0.430 | 0.53 | 0.128 |
| DLPFC 4 x 1 focal | Occipital GM R | 0.017 | 0.017 | 0.017 | -0.0001 | [-0.0002, 0.0000] | -0.510 | -0.49 [1.17] | -2.70 to 1.90 | 0.023 | 0.026 | 0.49 | 0.283 |
| DLPFC 4 x 1 focal | Frontal WM L | 0.331 | 0.352 | 0.367 | -0.0171 | [-0.0243, -0.0154] | -1.000 | -5.72 [1.94] | -10.09 to -2.12 | <0.001 | <0.001 | 0.03 | 0.983 |
| DLPFC 4 x 1 focal | Frontal WM R | 0.101 | 0.106 | 0.112 | -0.0057 | [-0.0062, -0.0052] | -1.000 | -5.17 [1.03] | -7.63 to -3.07 | <0.001 | <0.001 | 0.10 | 0.811 |
| DLPFC 4 x 1 focal | Parietal WM L | 0.118 | 0.125 | 0.133 | -0.0052 | [-0.0068, -0.0039] | -1.000 | -4.61 [2.23] | -9.05 to -1.27 | <0.001 | <0.001 | 0.51 | 0.140 |
| DLPFC 4 x 1 focal | Parietal WM R | 0.041 | 0.043 | 0.047 | -0.0021 | [-0.0024, -0.0015] | -0.995 | -4.20 [2.11] | -7.95 to 0.09 | <0.001 | <0.001 | 0.55 | 0.107 |
| DLPFC 4 x 1 focal | Temporal WM L | 0.109 | 0.113 | 0.122 | -0.0078 | [-0.0092, -0.0063] | -1.000 | -6.57 [2.02] | -11.04 to -2.79 | <0.001 | <0.001 | 0.23 | 0.616 |
| DLPFC 4 x 1 focal | Temporal WM R | 0.042 | 0.045 | 0.047 | -0.0027 | [-0.0032, -0.0022] | -1.000 | -5.90 [2.11] | -10.87 to -2.28 | <0.001 | <0.001 | 0.32 | 0.444 |
| DLPFC 4 x 1 focal | Occipital WM L | 0.031 | 0.033 | 0.034 | -0.0018 | [-0.0019, -0.0014] | -0.991 | -5.13 [2.43] | -9.48 to 1.06 | <0.001 | <0.001 | 0.59 | 0.211 |
| DLPFC 4 x 1 focal | Occipital WM R | 0.027 | 0.029 | 0.030 | -0.0015 | [-0.0018, -0.0012] | -0.972 | -4.84 [2.44] | -8.26 to 1.52 | <0.001 | <0.001 | 0.50 | 0.180 |
| DLPFC 4 x 1 focal | Whole brain | 0.169 | 0.170 | 0.170 | 0.0000 | [-0.0002, 0.0003] | 0.012 | -0.01 [0.49] | -1.21 to 1.15 | 0.955 | n/a | 0.46 | 0.283 |

**S-Table 4.** Surface metrics on the cortical middle surface, for the eight GM lobes under each of the four montages; the WM lobes have no middle surface. Δ nE is the change in the region mean of the E-field component normal to the surface, as a percentage of the region mean absolute normal component under the second model of the pair. Mean |Δ nE| is the mean absolute pointwise difference in the normal component, and RMS |E| the root-mean-square pointwise difference in E-field magnitude, each as a percentage of the same quantity under the second model. The upper block compares the two anisotropic models, the lower block DTI against the isotropic model. Cells are the cohort median with the interquartile range in brackets.

| Region | M1 bipolar | | | DLPFC bipolar | | | M1 4 x 1 focal | | | DLPFC 4 x 1 focal | | |
| --- | --- | --- | --- | --- | --- | --- | --- | --- | --- | --- | --- | --- |
|  | Δ nE | Mean \|Δ nE\| | RMS \|E\| | Δ nE | Mean \|Δ nE\| | RMS \|E\| | Δ nE | Mean \|Δ nE\| | RMS \|E\| | Δ nE | Mean \|Δ nE\| | RMS \|E\| |
| **MD-dMRI versus DTI** | | | | | | | | | | | | |
| Frontal GM L | 0.53 [-0.33, 0.99] | 6.95 [6.49, 7.63] | 4.47 [4.17, 4.70] | -0.48 [-0.79, 0.50] | 7.35 [6.94, 8.09] | 5.19 [4.91, 5.66] | 0.35 [0.03, 1.04] | 7.13 [6.61, 7.67] | 4.56 [4.26, 4.88] | -0.63 [-1.15, 0.19] | 6.60 [6.28, 7.15] | 4.67 [4.28, 5.07] |
| Frontal GM R | 0.31 [-0.16, 0.85] | 6.74 [6.50, 7.08] | 4.28 [3.96, 4.60] | 0.01 [-0.34, 0.44] | 6.96 [6.56, 7.21] | 4.74 [4.32, 5.00] | -0.12 [-0.59, 0.74] | 7.95 [7.10, 8.57] | 5.46 [5.07, 6.12] | -0.69 [-1.28, 0.01] | 7.01 [6.48, 7.55] | 4.55 [4.37, 4.79] |
| Parietal GM L | -0.81 [-1.21, -0.15] | 7.52 [6.93, 7.96] | 4.70 [4.34, 5.02] | -0.63 [-1.17, -0.30] | 7.65 [7.33, 8.16] | 5.18 [4.80, 5.61] | -0.80 [-1.37, -0.21] | 7.06 [6.65, 7.64] | 4.22 [3.96, 4.45] | 0.50 [-0.75, 1.05] | 6.65 [6.33, 6.82] | 5.41 [4.88, 5.90] |
| Parietal GM R | -0.26 [-0.69, 0.31] | 7.23 [6.76, 7.82] | 4.33 [3.93, 4.70] | -0.26 [-0.71, 0.31] | 7.39 [6.82, 8.03] | 4.52 [4.10, 5.03] | -1.19 [-1.41, -0.39] | 7.73 [7.37, 8.50] | 5.30 [4.91, 5.49] | -0.58 [-1.24, 0.09] | 6.71 [6.31, 7.12] | 4.92 [4.37, 5.32] |
| Temporal GM L | -0.06 [-0.37, 0.48] | 7.87 [7.35, 8.34] | 4.56 [4.33, 4.80] | -0.86 [-1.20, -0.15] | 7.78 [7.49, 8.26] | 4.79 [4.60, 5.18] | -0.18 [-0.66, 0.64] | 7.11 [6.95, 7.43] | 4.72 [4.42, 5.04] | 0.20 [-0.72, 0.93] | 7.10 [6.70, 7.58] | 4.62 [4.35, 5.05] |
| Temporal GM R | 1.59 [0.79, 2.45] | 8.21 [7.79, 9.00] | 3.49 [3.22, 3.75] | 1.52 [0.78, 2.40] | 8.16 [7.51, 8.85] | 3.67 [3.45, 4.02] | 1.67 [0.59, 2.48] | 8.54 [7.68, 9.29] | 5.09 [4.69, 5.37] | 0.72 [0.09, 1.60] | 6.54 [6.33, 7.12] | 5.12 [4.83, 5.38] |
| Occipital GM L | -1.08 [-1.97, 0.51] | 7.96 [7.53, 8.79] | 4.57 [4.24, 4.87] | -1.26 [-2.34, -0.06] | 8.33 [7.93, 8.60] | 4.63 [4.29, 4.89] | -2.33 [-2.96, -1.62] | 8.20 [7.63, 9.05] | 4.55 [4.31, 4.84] | -1.84 [-2.38, -1.27] | 7.73 [7.06, 8.23] | 4.85 [4.53, 5.22] |
| Occipital GM R | -0.24 [-1.06, 0.54] | 7.91 [7.32, 8.73] | 3.73 [3.56, 4.29] | -0.55 [-1.35, 0.32] | 8.15 [7.62, 9.08] | 4.34 [3.99, 5.09] | -1.61 [-2.38, -1.14] | 8.40 [8.06, 9.48] | 4.99 [4.31, 5.70] | -0.36 [-1.50, 0.44] | 7.52 [6.98, 7.99] | 4.37 [4.13, 4.67] |
| **DTI versus isotropic** | | | | | | | | | | | | |
| Frontal GM L | -0.25 [-0.75, -0.06] | 7.98 [7.75, 8.58] | 6.70 [6.35, 6.97] | 0.94 [0.53, 1.46] | 8.89 [8.57, 9.50] | 7.77 [7.40, 8.21] | -0.40 [-0.78, 0.04] | 8.48 [8.14, 9.01] | 7.15 [6.82, 7.47] | 1.34 [1.07, 1.71] | 7.84 [7.34, 8.43] | 6.28 [5.96, 6.71] |
| Frontal GM R | -1.32 [-1.53, -0.87] | 7.59 [7.15, 8.10] | 5.57 [5.30, 6.06] | -0.78 [-0.97, -0.23] | 7.75 [7.41, 8.33] | 6.25 [5.88, 6.79] | -0.74 [-0.89, -0.45] | 10.17 [9.30, 11.01] | 8.97 [8.21, 9.32] | 0.83 [0.57, 1.17] | 8.11 [7.63, 8.66] | 6.34 [5.80, 6.71] |
| Parietal GM L | 1.40 [1.02, 1.84] | 9.35 [8.94, 9.86] | 7.66 [7.39, 8.21] | 0.99 [0.39, 1.37] | 9.43 [9.03, 9.78] | 7.77 [7.57, 8.43] | 0.83 [0.41, 1.25] | 8.31 [8.10, 8.85] | 6.13 [5.86, 6.67] | -0.85 [-1.53, -0.48] | 7.57 [7.15, 8.05] | 6.47 [6.06, 6.71] |
| Parietal GM R | 0.78 [0.58, 1.04] | 8.98 [8.45, 9.41] | 6.67 [6.39, 6.98] | 0.62 [0.23, 0.92] | 9.11 [8.68, 9.41] | 6.93 [6.62, 7.42] | 0.78 [0.64, 1.16] | 10.44 [9.82, 10.89] | 8.59 [8.07, 9.38] | 0.59 [0.23, 0.92] | 8.00 [7.65, 8.36] | 6.24 [6.00, 6.48] |
| Temporal GM L | 0.49 [0.01, 1.08] | 8.11 [7.53, 8.56] | 5.98 [5.77, 6.43] | 1.19 [0.78, 1.41] | 8.15 [7.83, 8.51] | 6.49 [6.25, 6.81] | -0.51 [-0.73, -0.12] | 7.34 [7.07, 7.71] | 5.82 [5.65, 6.27] | -1.51 [-2.11, -0.81] | 6.81 [6.41, 7.11] | 4.86 [4.60, 5.13] |
| Temporal GM R | -2.88 [-3.12, -2.18] | 7.65 [7.20, 8.30] | 3.84 [3.43, 4.29] | -3.17 [-3.44, -2.65] | 7.74 [7.35, 8.44] | 4.18 [3.69, 4.34] | -2.76 [-3.34, -2.25] | 9.91 [9.07, 11.55] | 7.40 [6.67, 8.03] | -1.63 [-1.94, -1.16] | 7.39 [6.90, 7.69] | 6.05 [5.68, 6.32] |
| Occipital GM L | 0.79 [0.34, 0.94] | 7.98 [7.44, 8.56] | 6.09 [5.75, 6.57] | 0.98 [0.75, 1.33] | 7.90 [7.29, 8.44] | 5.76 [5.55, 6.29] | 0.61 [0.05, 1.15] | 8.30 [7.86, 8.84] | 6.67 [6.40, 7.13] | 0.54 [0.01, 1.54] | 7.21 [6.78, 7.92] | 6.07 [5.81, 6.45] |
| Occipital GM R | -0.42 [-0.67, -0.17] | 7.18 [6.87, 8.00] | 4.87 [4.46, 5.33] | -0.52 [-0.78, -0.31] | 7.31 [6.94, 7.99] | 5.15 [4.76, 5.59] | -0.25 [-0.50, 0.50] | 8.16 [7.79, 8.71] | 5.96 [5.71, 6.31] | 0.11 [-0.33, 0.46] | 7.05 [6.63, 7.28] | 5.29 [4.99, 5.73] |

**S-Table 5.** Per-region divergence between the principal diffusion direction (V1) of the MD-dMRI mean tensor and that of a DTI tensor, for the eight WM and eight GM lobes. The first angle column is against the separate DTI scan; the next four are against a DTI fit of the MD-dMRI data itself, over all linear-encoded volumes, over the b = 2000 s/mm² shell, and under each of two FA thresholds applied to the all-volume fit. Columns without a threshold keep every voxel in which both tensors are positive-definite, so the two thresholded columns are not comparable with the rest. FA difference is the fractional anisotropy of the mean tensor minus that of the separate DTI scan. Angle and FA cells are the cohort median with the interquartile range in brackets; where fewer participants contribute, the number is given in the cell. The last column correlates each region's median angle with the relative difference in p95 |E| between the models under the M1 bipolar montage, with a Fisher z 95% interval. That difference is negative in every participant and WM lobe, so a positive correlation means a larger angle went with a smaller difference in magnitude.

| Region | V1 angle (degrees) | | | | | FA difference, mean tensor minus DTI | Correlation with p95 \|E\| change, r [95% CI] |
| --- | --- | --- | --- | --- | --- | --- | --- |
|  | Between the two acquisitions | DTI fit, all volumes | DTI fit, b = 2000 shell | DTI fit, FA > 0.2 | DTI fit, FA > 0.3 |  |  |
| Frontal WM L | 17.7 [16.3, 18.7] | 11.9 [11.1, 13.4] | 13.5 [12.4, 15.0] | 7.9 [7.5, 8.9] | 6.3 [6.0, 7.0] | -0.067 [-0.081, -0.061] | 0.50 [0.16, 0.73] |
| Frontal WM R | 17.7 [16.0, 19.0] | 11.8 [11.2, 12.9] | 13.3 [12.6, 14.6] | 7.9 [7.2, 8.6] | 6.2 [5.8, 7.1] | -0.061 [-0.077, -0.052] | 0.64 [0.36, 0.82] |
| Parietal WM L | 17.6 [15.7, 19.3] | 11.5 [10.6, 12.0] | 12.7 [12.0, 13.3] | 7.9 [7.3, 8.6] | 6.5 [6.1, 7.3] | -0.081 [-0.097, -0.073] | 0.06 [-0.31, 0.42] |
| Parietal WM R | 17.9 [16.2, 19.3] | 11.4 [10.6, 12.2] | 12.5 [11.9, 13.6] | 7.8 [7.3, 8.2] | 6.2 [6.1, 6.8] | -0.083 [-0.097, -0.066] | 0.51 [0.18, 0.74] |
| Temporal WM L | 22.1 [20.5, 23.6] | 16.0 [15.2, 17.3] | 18.2 [17.3, 19.5] | 9.8 [9.4, 10.7] | 7.4 [6.9, 8.1] | -0.055 [-0.074, -0.046] | 0.46 [0.11, 0.71] |
| Temporal WM R | 22.6 [20.6, 23.6] | 16.3 [15.4, 17.2] | 18.4 [17.8, 19.5] | 9.6 [9.0, 10.4] | 6.8 [6.4, 7.5] | -0.052 [-0.068, -0.043] | 0.49 [0.15, 0.73] |
| Occipital WM L | 25.1 [23.0, 26.9] | 16.1 [14.9, 18.7] | 17.8 [16.7, 21.2] | 8.9 [8.1, 10.2] | 6.5 [6.1, 7.7] (n = 28) | -0.064 [-0.074, -0.054] | -0.04 [-0.40, 0.33] |
| Occipital WM R | 25.2 [23.9, 26.7] | 16.3 [15.1, 17.9] | 18.3 [16.7, 20.0] | 8.6 [7.5, 9.3] | 6.6 [5.7, 6.9] | -0.057 [-0.066, -0.044] | 0.26 [-0.12, 0.57] |
| Frontal GM L | 43.5 [42.3, 46.3] | 33.8 [32.1, 34.8] | 37.8 [35.9, 39.9] | 10.7 [9.5, 12.8] | no participant qualified | -0.001 [-0.005, 0.007] | -0.22 [-0.54, 0.16] |
| Frontal GM R | 43.9 [39.6, 45.0] | 31.9 [31.3, 34.1] | 36.6 [35.0, 39.0] | 10.5 [8.9, 12.0] (n = 27) | no participant qualified | -0.001 [-0.003, 0.004] | 0.06 [-0.31, 0.42] |
| Parietal GM L | 43.8 [41.5, 45.8] | 32.0 [30.9, 33.4] | 36.1 [34.2, 37.9] | 12.2 [10.2, 13.4] (n = 28) | no participant qualified | -0.004 [-0.006, 0.002] | 0.26 [-0.12, 0.57] |
| Parietal GM R | 42.9 [41.5, 45.2] | 32.4 [30.9, 34.1] | 36.4 [34.5, 37.6] | 11.2 [10.5, 12.9] | no participant qualified | -0.001 [-0.006, 0.003] | -0.28 [-0.58, 0.10] |
| Temporal GM L | 46.1 [44.7, 47.0] | 34.3 [33.1, 36.1] | 40.4 [38.6, 41.9] | 17.2 [14.4, 19.5] (n = 23) | no participant qualified | 0.022 [0.013, 0.028] | 0.02 [-0.35, 0.39] |
| Temporal GM R | 46.2 [44.0, 48.3] | 35.7 [33.3, 37.9] | 42.1 [39.3, 44.0] | 18.2 [14.7, 21.7] (n = 15) | no participant qualified | 0.022 [0.019, 0.032] | 0.27 [-0.11, 0.58] |
| Occipital GM L | 48.7 [46.4, 50.3] | 35.0 [31.8, 37.8] | 39.5 [37.2, 43.9] | 24.4 [19.1, 31.0] (n = 18) | 28.0 (n = 1) | 0.027 [0.022, 0.031] | 0.14 [-0.24, 0.48] |
| Occipital GM R | 47.0 [44.6, 49.9] | 35.3 [32.8, 37.0] | 41.4 [38.4, 42.6] | 26.6 [19.2, 30.5] (n = 19) | 43.2 (n = 1) | 0.029 [0.021, 0.035] | 0.45 [0.10, 0.70] |
